# A Truncated Serpin D1 Variant Regulates Adseverin-Dependent Actin Severing and Is Required for Osteoclastogenesis

**DOI:** 10.3390/cells15171607

**Published:** 2026-09-03

**Authors:** Yongqiang Wang, Chenfan Ji, Andrew Y. Wang, Masoud Norouzi, Isabel Ding, Michael Glogauer, Mark Abovsky, Igor Jurisica, Christopher A. McCulloch

**Affiliations:** 1 Faculty of Dentistry, University of Toronto, Toronto, ON M5G 1G6, Canada; yongqiang.wang@utoronto.ca (Y.W.); cji2027@meds.uwo.ca (C.J.); wanga6@myumanitoba.ca (A.Y.W.); masoud.norouzi@utoronto.ca (M.N.); isabel.xtong@gmail.com (I.D.); michael.glogauer@dentistry.utoronto.ca (M.G.); juris@ai.utoronto.ca (I.J.); 2 Osteoarthritis Research Program, Division of Orthopedic Surgery, Schroeder Arthritis Institute and Data Science Discovery Centre for Chronic Diseases, Krembil Research Institute, University Health Network, Toronto, ON M5T 0S8, Canada; mabovsky@yahoo.com; 3 Departments of Medical Biophysics and Computer Science, University of Toronto, Toronto, ON M5G 1L7, Canada; 4 Institute of Neuroimmunology, Slovak Academy of Sciences, 845 10 Bratislava, Slovakia; 5 Biological and Life Sciences Division, School of Digital Public Health, Mohamed bin Zayed University of Artificial Intelligence, Masdar City, Abu Dhabi 200120, United Arab Emirates

**Keywords:** cell fusion, actin severing, signaling, actin filaments

## Abstract

**Highlights:**

**What are the main findings?**

- The actin severing activity of adseverin is regulated by a truncated, intracellular form of Serpin D1 that is expressed in osteoclasts and that is increased by TNF-α.
- Deletion of the truncated form of *Serpind1* in cultured monocytes reduced the formation of osteoclasts.

**What are the implications of the main findings?**

- If shown to be required for the formation of osteoclasts at inflamed sites in intact animals, inhibition of the intracellular, truncated form of Serpin D1 could become a target for reducing osteoclast-dependent bone loss at inflammatory sites.
- Identifying novel regulators of monocyte fusion, such as restricting sub-cortical actin assembly in osteoclast precursor cells, could suggest new additional targets for limiting the formation of active osteoclasts.

**Abstract:**

Adseverin is a Ca^2+^-dependent, actin severing protein that promotes fusion of osteoclast precursors in osteoclast formation. Currently it is not understood how actin severing activity is regulated in osteoclastogenesis. Mass spectrometry of adseverin immunoprecipitates from osteoclasts showed that adseverin associated with Serpin D1. Purified Serpin D1 interacted with adseverin in vitro in a Ca^2+^-dependent manner. Ca^2+^ increased actin severing by ~2-fold. Cells that differentiated into osteoclasts expressed an intracellular, truncated *Serpind1* isoform that lacked exons 1 and 2, and part of exon 3. This truncated Serpin D1 isoform was distinct from the secreted, full-length Serpin D1 protein that inhibits thrombin activity. The expression of truncated Serpin D1 in RAW264.7 cells appeared to be further increased following TNF-α treatment during RANKL-induced osteoclastogenesis. CRISPR/Cas9-mediated partial deletion of exon 3 of *Serpind1*, which resulted in *Serpind1* knockout, increased the abundance of subcortical actin filaments. This treatment also altered the spatial distribution of adseverin, inhibited the expression of osteoclast-specific genes and reduced the formation of multinucleated osteoclasts by >90% in vitro. We conclude that a truncated, intracellular variant of Serpin D1 interacts with adseverin to promote actin severing and osteoclast formation.

## 1. Introduction

Osteoclasts are bone-resorbing cells that are crucial for bone development and skeletal homeostasis. These cells also play a central role in the dysregulated resorption of bone seen in inflammatory diseases such as rheumatoid arthritis and periodontitis. When activated, osteoclasts reside in resorptive pits on remodeling bone surfaces [1]. Osteoclasts are derived from monocyte/macrophage lineage precursor cells that initially form pre-fusion osteoclast progenitors. These progenitor cells subsequently fuse to form multinucleated osteoclasts [2,3]. In intact animals, osteoclast precursors undergo clonal expansion to form mature osteoclasts [4,5]. In vitro, osteoclasts can be generated by treating precursor cells with the receptor activator of nuclear factor kappa B ligand (RANKL) [6,7] and macrophage colony stimulating factor (M-CSF) [8]. During early stages of culture, pre-fusion osteoclasts migrate and come into contact with one another. Their subsequent fusion is facilitated by plasma membrane fusion proteins [9,10] and by inositol-1,3,5 trisphosphate (InsP_3_)-mediated Ca^2+^-release from the endoplasmic reticulum [11,12,13].

Close apposition or direct contact of adjacent, pre-fusion osteoclast precursor cells does not necessarily lead to fusion and osteoclast formation [10]. But one common feature of fusing pre-osteoclasts is their assembly of sub-cortical actin filaments. This process is essential for the formation of pseudopods that are required for RANKL-mediated fusion of osteoclast precursor cells [10,14]. In addition, actin assembly is necessary for the formation of podosomes, podosome clusters, actin rings and actin belts [10]. These structures are involved in the adhesion and migration of pre-fusion osteoclasts [15].

Actin assembly is a critical, rate-limiting process for osteoclastogenesis. In mammalian cells, actin assembly is mediated in part by a large array of actin binding proteins, which can cap, sever and cross-link actin filaments into larger, more complex structures. One of the actin binding proteins that mediates monocyte fusion in osteoclast formation is adseverin (Ads). The expression of Ads is increased during osteoclastogenesis [16,17]. Ads contributes to the formation of sub-cortical actin filaments, which are essential for cell fusion. Genetic deletion of Ads is not associated with any prominent phenotypic changes in developing mice (i.e., animals without inflammatory disease) [18]. But Ads-knockout (KO) mice do not exhibit increased osteoclastogenesis at sites of inflammation and are protected from inflammation-induced bone loss [19]. These data are consistent with findings in rheumatoid synovium, in which pro-inflammatory mediators strongly promote osteoclastogenesis by increasing the expression of RANKL and TNF-α in stromal/osteoblastic cells or lymphoid cells [20,21]. Accordingly, we consider that Ads is involved in the increased osteoclastogenesis seen at inflamed sites. Currently, it is not known how the actin filament modifying properties of Ads are regulated in osteoclastogenesis. It is also not understood how Ads-interacting proteins contribute to the generation of functional osteoclasts.

Here we used tandem mass spectrometry to identify Ads-associated proteins that contribute to osteoclastogenesis. One of the Ads-associated proteins we discovered is a truncated variant of Serpin D1, which is a secreted plasma serine protease inhibitor that regulates blood clotting by inhibiting thrombin [22,23]. Previous reports of mouse osteoclast precursor cells identified two sets of *Nfatc1*-regulated genes [24]. One of these genes was *Serpind1* [25]. *Serpind1*-deficient mice developed at Washington University (St. Louis, MO) with a complete deletion of exon 2 (which contains the ATG translation start site for the full-length protein), showed no loss of viability of homozygotic mice. In these mice there were no obvious alterations of normal bone development compared with wild-type (WT) mice [25,26]. However, in a different *Serpind1* KO mouse line developed at the University of Tokushima, Japan, in which a 120 bp fragment of exon 2 including the ATG site was deleted, the homozygotes were embryonic lethal. The viable heterozygote mice produced at Tokushima exhibited intimal hyperplasia after vascular stress compared with WT controls [27]. This finding was consistent with a reduction of Serpin D1, a known regulator of blood clotting. Assessments of the Tokushima homozygous KO mice were not conducted because of their embryonic lethality. Accordingly, the effects of *Serpind1* KO on osteoclast differentiation and bone phenotypes were not fully evaluated in these mice. Further, no reports have examined the role of Serpin D1 in Ads-dependent osteoclastogenesis. As Ads appears to regulate osteoclastogenesis only at bony sites affected by inflammation, such as in periodontitis [19], we examined the interaction between Serpin D1 and Ads in osteoclasts induced by RANKL alone or by RANKL in combination with the pro-inflammatory cytokine TNF-α. Our aim was to determine the role of truncated Serpin D1 in osteoclastogenesis, the underlying mechanisms involved and the relative importance of the pro-inflammatory cytokine, TNF-α.

## 2. Materials and Methods

### 2.1. Animals

A *Serpind1* KO exon 2 (E2) mouse strain was generated by Drs. Ken-ichi Aihara and Toshio Matsumoto at Tokushima University, Japan. Their cryopreserved embryos were used to generate *Serpind1* +/– mice. The Tokushima *Serpind1* mutant mouse strain was originally created by deleting a 120-bp fragment in exon 2 (including the ATG start site) and by inserting a phosphoglycerate kinase–neomycin cassette with a polyA termination signal. This mouse strain was maintained on an inbred background (C57BL6/J) after 10 generations of backcrossing [27]. The frozen embryos obtained from Tokushima were recovered by the Centre for Phenogenomics (TCP, owned and operated by Mt. Sinai Hospital and the Hospital for Sick Children, Toronto, ON, Canada). Mice were bred and housed in the Animal Resources Centre of the Ontario Institute for Cancer Research or in the Department of Comparative Medicine, University of Toronto, in controlled, pathogen-free environments.

As the Tokushima *Serpind1* homozygous KO (exon 2, E2) are embryonic lethal, *Serpind1* heterozygous KO (E2) and WT mice were crossed (by Taconic Biosciences, Rensselaer, NY, USA) to produce litter- and sex-matched heterozygous KO and WT mice for comparisons. The genotype of the mice was identified with genomic DNA obtained from ear notches or tail clips by polymerase chain reaction (PCR), with a pair of primers 5′-CTTCCTCGTGCTTTACGGTA-3′ (forward) and 5′-GTAGCCTGGTCCTTCAGGACTCGG-3′ (reverse). The PCR conditions were 35 cycles of denaturation for 30 s at 94 °C, annealing for 30 s at 54 °C, and extension for 45 s at 72 °C. Mice exhibiting a 740-bp band were considered *Serpind1* KO (E2). The animal protocol used in this particular study was also reviewed and approved by the Animal Care Committees of the University Health Network and the University of Toronto as described above and in accordance with policies and procedures formulated by the University Toronto Animal Ethics & Compliance Unit, and the Guide for the Care and Use of Laboratory Animals Research Statutes, Ontario (1980).

We endeavored to assess whether a specific deletion of the truncated *Serpind1* isoform could be created in C57 Bl/6 mice to enable study of the role of this *Serpind1* isoform in osteoclasts and the resultant bone phenotypes. We generated LoxP-inserted mice in which exon 3 of *Serpind1* was floxed. As described above, exon 3 corresponds to the transcriptional start site of the truncated *Serpind1* isoform that is specifically expressed in osteoclasts. These mice were intended to be crossed with a Cre recombinase line driven by the cathepsin K (*Ctsk*) promoter to achieve conditional knockout of *Serpind1* in osteoclasts. Four pairs of heterozygous LoxP-floxed mice were used as breeders and produced a total of 76 pups. Ear notch samples were collected for genotyping using two pairs of primers designed to detect LoxP insertion at both the distal and proximal sites flanking exon 3. (PCR primers: Genotyping premiers. F3: 5′-ATGAGATGATACAAGAAAGGTGCC-3′paired with R5: 5′-GTGATTCTCTAGCATTTGGCAGGA-3′; F4:5′-TAAGCTATGCCTGTTTATTTCAGCC-3′ paired with R6: 5′-GCAGACTGAGGCTATTCCTAAGAT-3′).

### 2.2. Osteoclast Differentiation and Resorption Assays

Mouse primary bone marrow cells or RAW264.7 cells were utilized as precursors for studies of osteoclast differentiation [28]. For isolation of primary bone marrow cells, marrow was extracted from tibia and femurs of 2-month-old mice. Briefly, femora and tibias were aseptically excised and carefully cleaned of any adherent soft tissue. Bone ends were cut and the marrow was flushed out, passed through a 20-gauge needle to break up remaining clumps, and single cell suspension were obtained. Cell suspensions (6 mL) were gently layered over 6 mL of Ficoll-Paque PLUS (Amersham Biosciences, Uppsala, Sweden) and centrifuged at 350× *g* for 30 min at room temperature (RT). The cell layer at the solution interface enriched in monocytes was collected with a sterile Pasteur pipette. Cells were washed twice with basic α-MEM, counted before the second wash and resuspended in complete α-MEM, which contains 10% fetal bovine serum (FBS) and antibiotics (164 IU/mL of penicillin G, 50 µg/mL of gentamicin, and 0.25 µg/mL of fungizone). Cells were seeded onto 60-mm Petri dishes (1 × 10^6^ cells/dish) for total RNA extraction (RT-PCR/qPCR), or cell lysate preparation (Western blot), or onto 8-well chamber slides (1× 10^4^ cells/well) for osteoclast differentiation assays. For these assays, complete α-MEM (4 mL per dish or 700 µL per well) was supplemented with 20 ng/mL M-CSF (PeproTech) and 60 ng/mL soluble RANKL and incubated for 6 days. Cell culture medium was replaced every other day during the 6-day incubation period. Cells that were stimulated with M-CSF alone were used as controls.

For studies of osteoclast formation from RAW264.7 cells, passage 4 cells were treated with recombinant mouse soluble RANKL. Cell culture medium was prepared by adding FBS and antibiotics to Dulbecco’s Modified Eagle Medium (DMEM). Complete medium containing fresh RANKL was added at day 2 of culture. Osteoclast formation from primary bone marrow cells or RAW264.7 cells was confirmed by Tartrate-resistant acid phosphatase (TRAcP) staining. Slides with cells stained for TRAcP were imaged with a light microscope or scanned with Axio Scan Z1 (Carl Zeiss Microscopy GmbH, Jena, Germany). The procedure used for TRAcP activity assays was described previously [29].

For study of osteoclast function, day 6 bone marrow cell-derived osteoclasts in 6-well plates were detached with Versene (Gibco) and replated in a 24-well plate with ivory dentine discs attached to the bottom of the well. After two more days incubation with M-CSF and RANKL, cells attached on dentin were removed with 1:10 diluted 6% bleach. The discs were stained with 1% toluidine blue and 1% sodium borate (1:1) for 10 s to view resorption. All cell incubations were conducted at 37 °C in 5% CO_2_ in a humidified incubator.

### 2.3. Micro-CT

Right-side femurs were obtained from 5-month-old male mice (four WT and four *Serpind1* heterozygous KO mice) that were litter, age and sex-matched. Formalin-fixed femurs were scanned at 6.1 μm pixel resolution for trabecular analysis and at 11.6 µm for cortical analysis using Skyscan 1174. Raw images were reconstructed with NRecon and analyzed using CTAn. For trabecular bone analysis, a region of interest (ROI) was identified 1 mm proximal from the growth plate, which served as a landmark. A total of 100 slices were selected in the distal direction from that point. Interstitial areas were chosen and thresholds were established to distinguish between bone and non-bone. After threshold determination, bone mineral density (BMD) values were acquired.

Cortical area measurements were conducted at the mid-diaphysis, with an ROI comprising 100 slices. Two distinct methods for drawing the ROI were employed: the shrink-wrap method and the donut method. The “shrink wrap” method involves automatic segmentation of cortical bone, fitting the ROI precisely to the outer edge of cortical bone in a slice-by-slice manner. Conversely, the “donut” method requires manual segmentation of cortical bone. The shrink-wrap method provides accurate values for Bone Volume to Tissue Volume (BV/TV), while the donut method facilitates precise measurement of closed porosity. The 2D slices were extracted from the shrink-wrap ROI, and the BMD of cortical bones was also measured.

### 2.4. Histology

Right-side distal tibiae were collected from four *Serpind1* heterozygous KO (E2) and four litter-matched WT male mice (8-weeks-old). Samples were fixed in 4% paraformaldehyde (PFA)/phosphate buffered saline (PBS) overnight and decalcified in a buffer containing 12.5% ethylenediaminetetraacetic acid (EDTA), 0.5% PFA, pH7.4, at 4 °C with rotation. The buffer was changed every other day for 21 days. Samples were embedded in paraffin, sectioned and prepared for TRAcP staining.

### 2.5. Mass Spectrometry

RAW264.7 cells (passage 4) were incubated in 25 mL of DMEM in 20 cm^2^ Petri dishes for 2 h to allow cell attachment. Cells were stimulated with RANKL (10 ng/mL) for 4 days, with or without TNF-α (30 ng/mL, PeproTech, Rocky Hill, NJ, USA). The medium was changed at day 2; after washing with pre-cooled sterilized PBS. Cells were lysed in 3 mL RIPA buffer (Sigma, St. Louis, MO, USA) containing phenylmethylsulfonyl fluoride (PMSF) and BD BaculoGold™ Protease Inhibitor Cocktail. Lysates were cleared by centrifugation at 4 °C for 5 min at 16,100× *g* to remove debris. The concentration of protein in the total cell lysate was measured with the Pierce BCA protein assay kit (Thermo Fisher Scientific, Waltham, MA, USA). Lysates (500 mg/sample) were incubated at 4 °C overnight with rotation in 1 mL RIPA buffer containing 5 mg protein A beads that were pre-equilibrated with a buffer (20 mM NaH_2_PO_4_·H_2_O and 150 mM NaCl; pH 8.0) with anti-gelsolin (1 µL) or anti-Ads (1 µL) antibodies to immunoprecipitate gelsolin or Ads, respectively. Rabbit IgG polyclonal antibody control (2 µL, Millipore-Sigma Cat. 12-370) was used as the immunoprecipitation control antibody. Beads were extensively washed with equilibration buffer, re-suspended in 150 µL buffer containing 50 mM Tris HCl and 100 mM NaCl (pH 8.0). A fraction of the bead-associated proteins was examined by Western blot. The remainder was digested with trypsin (15 µg, Sigma) in 0.01% formic acid at 37 °C O/N with rotation and the reaction was stopped with 1 µL 0.1% acetic acid. Tryptic digests were analyzed by tandem mass spectrometry (SPARC, Hospital for Sick Children, Toronto, ON, Canada) using Scaffold 5 software ver. 5.3.3 (Proteome Software, Portland, OR, USA).

### 2.6. ImmunoprEcipitation and Immunoblotting

Wild-type RAW264.7 cells (2 × 10^6^; passage 6) were treated with RANKL (60 ng/mL) and TNF-α (30 ng/mL) in 20 mL DMEM containing 10% FBS and antibiotics for two days in 10 cm dishes. The medium was replaced with fresh complete DMEM containing RANKL and TNF-α at the same concentrations. Lysates were prepared as described above (Mass Spectrometry section) and were centrifuged in a micro-centrifuge for 5 min at 4 °C to remove cell debris. Total protein concentrations were measured using BCA assay.

For immunoprecipitation experiments, protein A Sepharose beads (Sigma P3391, 5 mg) were incubated in 200 µL RIPA buffer for 30 min at RT. The beads were incubated with rotation for an additional hour at RT in 1 mL RIPA buffer containing either anti-Ads antibody (2 µL) or rabbit IgG polyclonal antibody control (2 µL, Millipore-Sigma Cat. 12-370). After 3× washes in PBS, the beads were incubated at 4 °C O/N in 1 mL RIPA buffer containing 500 µg total cell lysate and 1 mM CaCl_2_. The beads were washed 6 times with 1 mL ice-cold PBS containing PMSF in each wash and boiled in 80 µL 1% SDS lysis buffer. After centrifugation, 20 µL/lane was loaded on to 10% SDS-PAGE gels for separation and transfer to nitrocellulose membranes. Membranes were blocked with 1% bovine serum albumin (BSA) or 5% skim milk in Tris-Buffered Saline with 0.05% Tween-20 (TBST) for one hour and incubated in the same buffer containing 0.4 µg/mL goat anti-mouse Serpin D1 antibody (R&D, Minneapolis, MN, USA, 0.15 mg/mL) or goat anti-human Heparin Cofactor-II antibody (Affinity Biologicals, Ancaster, ON, Canada, 1:2000) at 4 °C overnight. Membranes were washed with TBST and incubated with IRDye^®^ 800CW donkey anti-goat IgG antibody in 5% milk/TBST for one hour. Rabbit anti-mouse Ads antibody (1:20,000 diluted in 5% milk/TBST) was used for immunoblotting to assess Ads abundance in whole cell lysates.

### 2.7. Bead Pull-Down Assays and Surface Plasmon Resonance

The interaction between Ads and either full-length or alternatively transcribed *Serpind1* was investigated. For study of Ads and the full-length Serpin D1 interaction, full-length mouse Serpin D1 (Cat. no. 1265-PI, R&D, Minneapolis, MN, USA) was used. Ads-bound Sepharose beads were prepared by transformation of *E. coli* (BL21) with a glutathione S-transferase (GST)-Ads expression plasmid construct [29]. Transformed bacteria were incubated overnight at 37 °C in 100 mL lysogeny broth medium supplied with 100 µg/mL ampicillin. On the next day, Ads expression was induced by 1 mM isopropyl β-D-1-thiogalactopyranoside (IPTG) at 25 °C for 2–3 h. Bacterial cells were pelleted (6000 g for 15 min at 4 °C) and the pellet was re-suspended in STE buffer (150 mM NaCl, 20 mM Tris pH 8.0, 1 mM EDTA) containing a proteinase inhibitor cocktail and PMSF. The suspension was treated with 1 mg/mL lysozyme at RT for 15 min. Triton X-100 (final concentration 1%) was added to the suspensions, which were sonicated on ice. After sonication, lysate was treated with benzonase (25 units/mL, Sigma) on ice for 30 min to reduce viscosity. Cell debris was removed by centrifugation of the samples at 12,500× *g*, 4 °C for 30 min. The supernatant was collected and incubated overnight at 4 °C with 1.5 mL Glutathione Sepharose 4B resin (GE Healthcare), which were washed, split into two tubes, and re-suspended in 5 mL buffer containing either Ca^2+^ or ethylene glycol-bis (β-aminoethyl ether)-N,N,N’,N′-tetra-acetic acid (EGTA) [30]. Beads (300 µL) were incubated with varying amounts of recombinant Serpin D1 protein at RT for 50 min. Beads were washed vigorously at least 6 times and eluted with 80 µL Tris-EDTA (TE) buffer (20 mM Tris pH 8.0 and 1 mM EDTA) containing 10 mM reduced glutathione. Laemmli buffer (20 µL of 5×) was added and the samples were denatured and separated with 10% polyacrylamide gels for Western blot analysis.

For study of interactions between Serpin D1 and Ads, a truncated fragment containing the alternatively transcribed *Serpind1* (M286-S478) was cloned into PGEX-4T and expressed in *E. coli*. This isoform of mouse *Serpind1* (starting from the first ATG site in exon 3) was amplified by using a primer pair: forward 5′-GGATCCCCAGGAATTATGACGCACAACCACAACTTCC-3′ and, reverse 5′-GATGCGGCCGCTCGATTAAGACTTGGCAGGGTTGG-3′. The expressed protein was purified with Glutathione Sepharose 4B resin (GE Healthcare) and dialyzed against PBS. GST purified from the empty vector was used as the control.

The interaction between purified Ads and Serpin D1 was assessed using Surface Plasmon Resonance (Nicoya Lifesciences, Kitchener, ON, Canada). Carboxyl sensors were used as a substrate for immobilization of the GST-tagged truncated Serpin D1 (M286-S478) ligand while various concentrations of Ads analyte were injected. GST alone was used as a negative control.

### 2.8. Computational Modeling

Protein interaction prediction was performed using IID ver. 2025-05 (https://ophid.utoronto.ca/iid; accessed on 20 June 2025) [31]. The structure of the two mouse proteins (Serpin D1 (P49182) and Ads (Q60604)) was modeled using AlphaFold version 2 Protein Structure Database (https://alphafold.ebi.ac.uk; accessed on 15 April 2025). Their interaction was modeled using the docking software MEGADOCK ver. 4.1.1 (https://www.bi.cs.titech.ac.jp/megadock/ppi.html; accessed on 26 March 2024) [32]. ChimeraX ver. 1.10.1 visualization software (https://www.rbvi.ucsf.edu/chimerax; accessed on 30 July 2025) [33] was used to visualize and interpret the docking results.

### 2.9. Proximity Ligation Assay (PLA)

Raw264.7 cells (passage 6, 1 × 10^4^) were plated into each well of an 8-well chamber slide. The cells were treated with 60 ng/mL RANKL for 5 days to promote the formation of multinucleated osteoclasts (OCs). Cell culture medium was changed on day 2 of treatment and the resulting osteoclasts were processed according to the Duolink^®^ Proximity Ligation Assay protocol. Briefly, cells were fixed with 4% PFA, permeabilized with 0.2% triton x-100 in PBS for 2 min, blocked with Duolink^®^ Blocking Solution, and incubated with rabbit anti-Ads (purified from serum of immunized rabbits, diluted 1:1000) and/or goat anti-Serpin D1 (R&D, AF1265, diluted 1:200) primary antibodies, and the corresponding secondary antibodies (anti-rabbit PLUS, Sigma, DUO92002) and (anti-goat MINUS, Sigma, DUO92006). Bound probes were ligated and amplified using the Duolink^®^ In Situ Detection Reagents Orange kit (Sigma, DUO92007). Samples were mounted in Duolink^®^ In Situ Mounting Medium with 4′-6-diamidino-2-phenylindole (DAPI) (Sigma, DUO82040) and imaged with a 40× oil-immersion objective on a Leica SP8 microscope. The proportion of signal (red dots) superimposed over cell areas and over equivalent areas on the slide without cells was counted for 50 cells in 3 different preparations and the mean ± standard deviations of these proportions were calculated.

### 2.10. Intracellular Ca^2+^ Imaging

As the differentiation of osteoclasts from monocytes is regulated in part by InsP_3_-mediated Ca^2+^-release from the endoplasmic reticulum (ER) [11,12,13], we imaged [Ca^2+^]_i_ by fura-2 and ratio fluorimetry. Cells (1 × 10^5^/dish) were incubated in 35-mm glass bottom dishes (MatTek, Ashland, MA, USA) containing 60 ng/mL RANKL for two days. Cell culture medium was replaced on day 2. On day 3 of imaging, the cells were washed once with pre-warmed PBS and incubated at 37 °C with 2 µg/mL fura-2/AM in one mL of serum-free DMEM. The medium was then replaced with one mL of live cell imaging buffer containing 140 mM NaCl, 2.5 mM KCl, 1.8 mM CaCl_2_, 1.0 mM MgCl_2_ and 20 mM HEPES (Thermo Fisher), supplemented with 1% FBS and 60 ng/mL RANKL, and incubated at 37 °C for 30 min. Ca^2+^ imaging was conducted on a Nikon Ratiometric fluorimeter microscope with a 40× objective and with analysis by SimplePCI software (Version 6.6, Hamamatsu Corporation, Bridgewater, NJ, USA).

### 2.11. CRISPR/Cas9

The plasmid vector pSpCas9n(BB)-2A-Puro (PX462) V2.0 (Addgene, Watertown, MA, USA, 1 µg) was incubated with 1 µL Bbsl-HF (NEB R3539S, Ipswich, MA, USA) and 1 µL alkaline phosphatase in 1× CutSmart buffer (B7204S) for 30 min (20 µL reaction volume). The linearized and dephosphorylated plasmid vector was gel purified. Complementary top and bottom oligonucleotides encoding two pairs of guide RNAs targeting mouse *Serpind1* exon 3 were synthesized (ACGT Corp., Toronto, ON, Canada). The sequences of the oligonucleotides were: gRNA1-exon 3-top 5′-caccgAACTAAAGGGAATTTCCTTG-3′ and gRNA1-exon 3-bottom 5′-aaacCAAGGAAATTCCCTTTAGTTc-3′; and gRNA2-exon 3-top 5′-caccgCTACTTCTCTCTCATTCAGC-3′ and gRNA2-exon 3-bottom 5′-aaacGCTGAATGAGAGAGAAGTAGc-3′). Mouse *Serpind1* gRNA stock solutions (1 µL of 100 µM) were phosphorylated using T4 polynucleotide kinase (0.5 µL) and annealed, respectively in a thermocycler (initial incubation step at 37 °C for 30 min, followed by 95 °C for 5 min, 72 °C for 2 min, 37 °C for 2 min and ended at 25 °C for 2 min, in a 10 µL reaction volume). The phosphorylated and annealed oligo duplexes (1 µL) were inserted into the linearized plasmid vector (50 ng) described above. The inserts in the resulting constructs were sequenced (ACGT Corp, Toronto, ON, Canada). RAW264.7 cells (1 × 10^6^, passage 6) were co-transfected with 2 µg of each of the two gRNA constructs using the Amaxa nucleofector^®^ system (program D-032) and a transfection buffer 1M [31]. Transfected cells (~10% of cells) were selected in 7 µg/mL puromycin one day after transfection for two days. Standard limiting dilution was performed to create single cell clones after antibiotic selection, which was confirmed by the Surveyor assay using primers (forward 5′-TTGGGGTTGGTCCTAGAGAG-3′ and reverse 5′-AGGAGGATCATGGCAGGTTT-3′) and high fidelity Phusion enzyme (NEB M0530S). For sequencing *Serpind1* in single cell clones, genomic DNA was purified with the Quick-gDNA MiniPrep kit and was used as the template for PCR which was conducted in 50 µL reaction volumes using Phusion enzyme and Surveyor forward and reverse primers. The PCR product was gel purified; 3’- A overhangs were added and cloned into the pGEM^®^-T Easy vector. Samples were sequenced (Centre for Applied Genomics; Hospital for Sick Children) with the Surveyor forward or reverse primers.

Two additional pairs of mouse *Serpind1*-targeting gRNA oligos were designed against exon 2 (E2). The sequences of the oligos were gRNA1-E2-top 5′-caccgTTTATGTGTATTGGGAGCAA-3′and gRNA1-E2-bottom. 5′-aaacTTGCTCCCAATACACATAAAc-3′; and gRNA2-E2-top 5′-caccgAGAGAAAGAAGAGTGCAGAG-3′ and gRNA2-E2-bottom 5′-aaacCTCTGCACTCTTCTTTCTCTc-3′. These sequences were cloned into the plasmid vector pSpCas9n(BB)-2A-Puro (PX462) V2.0. The primer sequences used for the Surveyor assay to confirm the deletion were: forward 5′-TAGCTGGTGTGAGCATTGGTG-3′ and reverse 5′-TGCCAACAGGTGCTATGAAGA-3′. All cell lines derived by limiting dilution were subjected to sequencing of the targeted exon 2 region.

### 2.12. Mutagenesis

A primer pair, forward 5′-aggcggtagcATGACGCACAACCACAACTTCCGGCTGAATGAGA-3′ and reverse, 5′-CGTCATgctaccgcctccacccttatcgtcgtcatccttgtaat-3′ was used to delete the entire *Serpind1* exon 2 and 29 nucleotides in exon 3 before the first ATG codon, (flanked by the lowercase and capital nucleotide sequences in the primers). We used the Sino Biological construct MG50121-NF as the template, and a modified mutagenesis method [32] based on the QuikChange Lightning Site-Directed Mutagenesis Kit (Cat no. 210518, Agilent Technologies, Santa Clara, CA, USA). The resulted plasmid construct expressing Flag-tagged, alternatively transcribed Serpin D1 (M286-S478) was used to assess Serpin D1/Ads interactions.

### 2.13. Retroviral Rescue of Serpind1-Deficient Cells for Osteoclastogenesis

The truncated mouse *Serpind1* isoform containing a C-terminal FLAG tag was amplified from a commercially available *Serpind1* cDNA clone (Sino Biological, Beijing, China, MG50121-CF) using Q5 High-Fidelity DNA Polymerase and the following primers: forward, 5′-GCGCGCGGCCGCCGCCACCATGCTTATTGTAGTTCCACGGAAGCTATCG-3′; and reverse, 5′-GCGCATCGATTCATTACTTATCGTCGTCATCCTTGTAATCAGAGCCTCCA-3′. The resulting PCR product was cloned into the NotI/ClaI-linearized pLNCX2-N1 retroviral vector and verified by Sanger sequencing.

GP293 packaging cells, seeded in 6-well plates at 30–40% confluence, were co-transfected with pLNCX2-N1-truncated *Serpind1* and pVSV-G (2 μg of each plasmid per well in 2 mL culture medium) using FuGENE HD (Promega Corporation, Madison, WI, USA). Retroviral particles released into the culture supernatant were collected and used to transduce *Serpind1* exon 3-deleted CRISPR clone 2 cells. G418-resistant cells, selected using 800 μg/mL G418, were used as stably transduced cells. For osteoclastogenesis, cells were seeded in 8-well chamber slides at 1 × 10^4^ cells/well in 700 μL complete DMEM supplemented with 60 ng/mL RANKL. Cells were cultured for 4 days, with medium replacement on day 2, then fixed in 10% formalin for 5 min and subjected to TRAP staining.

### 2.14. Rapid Amplification of cDNA Ends (RACE)

RAW264.7 cells (passage 5, 5 × 10^7^ cells/dish in 60-mm dishes) were used as models of osteoclast precursor cells and stimulated with RANKL (60 ng/mL) for 4 days to induce osteoclast formation; medium was refreshed on day 2. Total RNA was extracted using RNeasy Plus Mini kit (Qiagen, Germantown, MD, USA), followed by reverse transcription to cDNA. *Serpind1* expression in RAW264.7 cell-derived osteoclasts was evaluated with two primer pairs: primer pair 1 with forward 5′-GGCAAACAACCACATTCTG-3′ and reverse 5′-GGACCTCCACCAGGTTGTAA-3′ targeting exons 2 to 4; expected amplicon size 443 bp for full-length *Serpind1*; primer pair 2 with forward 5′-GAGTACGTAGGGGGCATCAG-3′ and reverse 5′-GGACCTCCACCAGGTTGTAA-3′ targeting exons 3 to 4; expected amplicon size 184 bp as described [25]. GAPDH (used as a loading control) was amplified with forward 5′-CCTTCCGTGTTCCTACCCC-3′ and reverse 5′-GCCCAAGATGCCCTTCAGT-3′ primers (amplicon size 131 bp, targeting exon 5 to 6) [29].

The 5′/3′ RACE 2nd generation kit (Roche, Indianapolis, IN, USA), which was designed for the rapid amplification of 5′ and 3′ cDNA ends, was employed to examine the start and end sites of *Serpind1* transcript in osteoclasts. For 5′-RACE, total RNA isolated from osteoclasts was reverse transcribed into the first-strand cDNA using a *Serpind1*-specific reverse primer. 5′-GGACCTCCACCAGGTTGTAA-3′ (1) located in exon 4. Subsequently, the synthesized cDNA was purified using Qiagen PCR purification kit (Qiaquick, Germantown, MD, USA, Cat. no. 28104). The 3′-end of the purified cDNA was extended with a homo-polymeric A-tail. Subsequently, dA-tailed cDNA underwent PCR amplification using a *Serpind1*-specific reverse primer. 5′-AAGTACCTCTCGAGTTCTGTTTGTCATGCT-3′ and oligo dT-anchor primer Vial 8 from the kit. The PCR product was gel purified and sequenced using the reverse primer as the sequencing primer. Alternatively, the PCR product was amplified using a *Serpind1*-specific reverse primer (5′-CCACCACCTGGGGTGTAAGCTGT-3′) and PCR anchor forward primer (Vial 9) from the kit, followed by sequencing with the reverse primer as the sequencing primer.

3′-RACE was performed using the oligo d(T)-anchor primer (Vial 8) from the kit for the reverse transcription. Subsequently, the synthesized cDNA was amplified with a *Serpind1*-specific primer 5′-ATGGTTGTGCCAGCTATTCC-3′ and the PCR anchor primer Vial 9 from the kit. The resulting PCR product was sequenced, utilizing 5′-TGCCCAGTGGGAAAGCAATC-3′ as the sequencing primer. All PCR reactions were conducted using the Q5 master mix (Ipswich, MA, USA) following a program consisting of denaturation at 98 °C for 30 s, followed by 35 cycles of 98 °C for 10 s, 68 °C for 30 s, and 72 °C for 30 s, with a final extension step at 72 °C for 2 min.

### 2.15. Promoter Activity

A pair of primers 5′-GCGCGAGCTCACCTGCTAAGTCACCTCTCTGTTCCTAAAT-3′ (forward) and 5′-GCGCCTCGAGATAGCTTCCGTGGAACTACAATAAGCATGC-3′ (reverse) was used to amplify a 2054 bp fragment, by using Q5 high-fidelity DNA polymerase and genomic DNA isolated from RAW264.7 cells. This fragment contains 1992 bp upstream of the transcription start site and 62 bp of the truncated isoform *Serpind1* transcript identified in mouse osteoclasts by 5′-RACE. The resulting PCR product was digested with SacI/XhoI and inserted into the linearized corresponding sites of the pGL4.10 [Luc2] vector (Promega, Madison, WI, USA, Cat. No. E6651). This construct (3 µg) was co-introduced into RAW264.7 cells (passage 6, 1.5 × 10^6^) with 150 ng Renilla internal control plasmid construct pRL-TK (Promega, Cat. No. E2241 (20:1), by using an Amaxa nucleofector (Walkersville, MD, USA, program D-032) with buffer 1M reagent [31]. The transfected cells were seeded into a 6-well tissue culture plate. RANKL (60 ng/mL) was added to cultures one hour later, after the cells attached to the wells. Transfected cells that were not stimulated with RANKL were used for comparisons. Non-transfected cells (treated with or without RANKL) were prepared to measure background signals relative to luminometer units (RLU) for correction. After 2 to 3 days incubation, luciferase activity was measured with a Lumat LB 9508 single tube luminometer according to the manufacturer’s protocol of dual-luciferase reporter assays (Promega, Cat. No. E1910).

### 2.16. Real-Time and Semi-Quantitative Reverse Transcription PCR

Total RNA was extracted from cells or liver tissue with the RNeasy mini kit (Qiagen). Fresh, isolated liver tissue was preserved in RNAlater (Thermo Fisher; an RNA stabilization reagent) and flash-frozen in liquid nitrogen before extraction. Real-time or semi-quantitative reverse transcription PCR (RT-qPCR) was performed to measure mRNA abundance using samples (1 µg) that were reverse-transcribed into cDNA with Superscript II reverse transcriptase (200 units; Invitrogen, Waltham, MA, USA) and Oligo-dT_18_ VN primers (1 µM, ACGT Corp.) in a 20 µL reaction volume. Quantitative real-time PCR was performed in triplicate using the BioRad CFX96 real-time system (Bio-Rad Laboratories, Hercules, CA, USA). Each 20 µL reaction contained 5 µL of 1:10 diluted cDNA, 200 nM of forward and reverse primers and 10 µL of the Advanced qPCR master mix (Wisent, Saint-Jean-Baptiste, QC, Canada). PCR conditions were as follows: 95 °C for 2 min followed by 40 cycles of 95 °C for 5 s and 60 °C for 25 s. Melt curve analysis (95 °C for 5 s, 65 °C for 5 s, and 65 °C to 95 °C with 0.5 °C increase every 5 s) of the amplified product was used to determine the specificity of the PCR reaction. The expression of mRNA was normalized to GAPDH. For semi-quantitative RT-PCR, the cDNA was amplified for 28 cycles. Primers used for mRNA analysis are described in Supplementary Table S1.

### 2.17. Analysis of Signal Peptide

We assessed the absence or presence of the Serpin D1 signal peptide. Total RNA was isolated from osteoclasts derived from RAW264.7 cells treated with RANKL. Samples from mouse liver tissue were used as the positive control for the full-length *Serpind1*. One microgram of RNA was reverse-transcribed using the iScript cDNA synthesis kit, and the resulting cDNA was diluted 1:10. Five microliters of the diluted cDNA were used as the template for PCR. Two primer sets were employed: SP-F1/E4R (forward: 5′-ATGAAACATCCACTCTGCACT-3′; reverse: 5′-GGACCTCCACCAGGTTGTAA-3′; amplicon size: 1162 bp) and SP-F2/E4R (forward: 5′-TGCACTCTTCTTTCTCTCATCA-3′; reverse: 5′-GGACCTCCACCAGGTTGTAA-3′; amplicon size: 1147 bp). Both F1 and F2 primers are located within the signal peptide-coding region of full-length *Serpind1*, to ensure the primers target this region specifically. E4R is a reverse primer located in Exon 4. Mouse GAPDH was amplified as an internal control (forward: 5′-CCTTCCGTGTTCCTACCCC-3′; reverse: 5′-GCCCAAGATGCCCTTCAGT-3′; amplicon size: 131 bp) and the primers targeting exons 3–4 (forward: 5′-GAGTACGTAGGGGGCATCAG-3′; reverse: 5′-GGACCTCCACCAGGTTGTAA-3′; amplicon size: 184 bp) (2) were used to detect both the full-length and truncated *Serpind1*.

### 2.18. Western Blot Analysis

Cells attached to dishes were lysed with RIPA buffer containing protease inhibitors and centrifuged to remove cell debris. Lysates (20 µg/lane) were mixed with Laemmli buffer and boiled. Western blot analysis was performed as described [29] using rabbit anti-mouse Ads antibody (1:20,000) produced by immunization of rabbits with mouse recombinant Ads protein expressed and purified in our laboratory. Mouse Ads cDNA [33] was used for production of the immunogen. For some experiments we used a separate Ads antibody (goat anti-mouse Serpin D1 antibody; 0.1 ug/mL, R&D Systems). In certain experiments, lysates from cells that were electroporated with *Serpind1* cDNA (in pCMV3-SP-N-Flag, Sino Biological, Catalog no. MG50121-NF) were used as a positive control for Serpin D1. For loading controls, immunoblots were stained with a β-actin antibody (Sigma; clone AC-15; 1:8000). Secondary antibodies were IRDye^®^ 800CW donkey anti-goat IgG (1:10,000) and IRDye^®^ 680RD goat anti-mouse IgG (1:10,000). Membranes were imaged with an Odyssey Fc detection system (Li-Cor, Lincoln, NE, USA). ImageJ (NIH) (Version 1.54, National Institutes of Health, Bethesda, MD, USA) or Image Studio™ Lite Quantification Software (LI-COR) (Version 5.2.5, LI-COR Biosciences, Lincoln, NE, USA) were used to quantify the optical density of protein bands from Western blots and immunoprecipitation reactions. The proportion of blot densities for Ads and for Serpin D1 were then computed followed by estimation of mean ± standard deviation.

### 2.19. Immunostaining and Colocalization

RAW264.7 cells (1.5× 10^4^/well in 8-well chamber slides) were differentiated into osteoclasts with RANKL (60 ng/mL) for 4 days. Cells were rinsed with pre-warmed PBS and fixed with 4% PFA in PBS for 20 min at RT. After three washes with PBS, cells were incubated in 100 mM glycine/PBS for 10 min. Cells were permeabilized with 0.1% Triton-100 in PBS for 5 min and blocked in 1% BSA/PBS for 30 min. Cells were incubated with an antibody to Ads (1:1000) in 1% BSA/PBS overnight at 4 °C. After three rinses, cells were sequentially stained with goat anti-rabbit IgG-488 (Invitrogen, 1:1000) for one hour at 37 °C in the dark, followed by rhodamine phalloidin (Sigma; 1:30) for 30 min at RT and counterstained with DAPI (Sigma; 0.0625 µM) for 10 min in dark. The fluorescence intensity of Ads and of cell diameters were measured by drawing a line across the cell body through the centroid (NIH ImageJ).

In experiments that involved analysis of colocalization, WT osteoclasts at day 4 were treated with 1 µg/mL brefeldin A for 2.5 h before fixation. Cells were immunostained as described above, and for Serpin D1 (antibody from R&D, 1:200) followed by secondary antibody (donkey anti-goat IgG, Alexa Fluor 647; Invitrogen, 1:500). Osteoclasts were generated from the wild-type RAW264.7-derived H10 cell line, which exhibits high osteoclastogenic potential, by treatment with 60 ng/mL RANKL for 4 days. The culture medium was refreshed on day 2. In some experiments, cultures were not treated with brefeldin A. Cells were fixed with 4% paraformaldehyde in PBS, quenched with 100 mM glycine, permeabilized with 0.1% Triton X-100 in 1% BSA/PBS, and blocked with 1% BSA in PBS for 1 h. Cells were then incubated overnight at 4 °C with the primary antibodies goat anti-mouse Serpin D1 (R&D Systems) and rabbit anti-mouse adseverin (homemade), at the same concentrations described above. Following primary antibody incubation, cells were stained for 1 h at 37 °C in the dark with the secondary antibodies Alexa Fluor 568 donkey anti-goat IgG (1:500; Invitrogen, A11057) and Alexa Fluor 647 donkey anti-rabbit IgG (1:500; Life Technologies, Carlsbad, CA, USA, A21244). Cells were then air-dried and mounted. Images were acquired using a Zeiss Airyscan confocal microscope, with Serpin D1 displayed in red and adseverin shown in green (pseudo-colored). Colocalization analysis of adseverin and Serpin D1 in multinucleated osteoclasts was conducted using Imaris and Pearson’s correlation coefficient was calculated.

For flow cytometry analyses, wild-type and *Serpind1*-KO (E3) RAW264.7 cells (0.5 × 10^6^/dish) were treated with RANKL (60 ng/mL) for 4 days in 10 mL complete DMEM. The medium was changed at day 2. Cells were washed with pre-warmed PBS and detached with pre-warmed Versene, fixed with chilled 70% ethanol for 20 min, washed with PBS containing 0.5% flow cytometry staining buffer, and resuspended in FACs buffer (1 × Phosphate Buffered Saline without Ca^2+^ or Mg^2+^, 1 mM EDTA, 1% bovine serum albumin) at 1 × 10^6^ cells/200 µL. Cell suspensions were transferred to a 96-well U-shaped-bottom plate (200 µL/well) and pelleted by centrifugation. Cells were incubated with primary antibody (rabbit anti-mouse Ads, 1:1000 in FACs buffer) for 1 h at 37 °C. After 3 washes with FACs buffer, cells were stained with goat anti-rabbit IgG conjugated to Alexa fluor^®^ 488 (1:1000) at 37 °C for 1 h in the dark. After washing, cells were resuspended in one mL FACs buffer and analyzed by flow cytometry. Cells were incubated with pre-immune serum as control. Each cell type was stained in triplicate wells.

### 2.20. Actin Severing

Pyrene-labelled, lyophilized rabbit muscle actin powder (Cytoskeleton, Cat. no. AP05, Denver, CO, USA) was resuspended with 50 µL of cold, distilled, and sterilized H_2_O. The reconstituted protein stock (465 µM) was further diluted to a final concentration of 0.3 µM with a polymerization buffer containing 150 mM KCl, 20 mM HEPES (pH 7.4), 0.5 mM ATP, 0.2 mM DTT, 2 mM MgCl_2_, and 0.2 mM CaCl_2_. The diluted protein was incubated at RT for 2 h in dark. The reduction in fluorescence (as quantified by photon counts) due to actin severing was measured for 500 s with an integration time of 1 s with a PTI fluorimeter (London, ON, Canada; excitation—365 nm, emission—386 nm). Gelsolin (Cytoskeleton, Cat. no. HPG6) at a final concentration of 0.10 µM, was used as the positive control for actin severing. GST-fused Ads (0.20 µM) was examined alone or with Serpin D1 (M286-S478) at a concentration of 0.10 µM in the co-incubation assays. The purified GST alone was used as the negative control. To quantify severing, the starting actin pyrene fluorescence and fluorescence obtained at 5 s after the addition of actin to Ads and/or other molecules were recorded. Ads and Serpin D1 were mixed and incubated for 10 min before actin addition. The experiments were performed 3 to 5 times for each condition. Photon counts were normalized to the initial counts before addition of reagents. The reduction of fluorescence was analyzed with the “R” program; these data were expressed as a rate constant k = −LN (A_500_/A_0_)/500, in which A_500_ and A_0_ are the photon counts 500 s after and before counting. The data from at least 3 different experiments were used to estimate the rate constant for each experimental group, which were then plotted.

### 2.21. Statistical Analysis

One-way analysis of variance (ANOVA) was used to compare differences between multiple groups (>2). Individual differences were assessed by Tukey’s *post hoc* test. For comparisons between two groups, Student’s *t*-test was used. Summary data are generally presented as mean ± standard deviation. In some figures as specifically indicated, the mean ± standard error of the mean are described in the accompanying legend. Statistical significance was set at *p* < 0.05, based in part on the finding of normally distributed data sets for most experiments, except as indicated in specific figures. For all experiments, 3 or more biological replicates were included in each analysis, except in those figures in which larger sample sizes were used as indicated.

## 3. Results

### 3.1. Serpin D1 Associates with Ads in Osteoclasts

**Figure 1:**
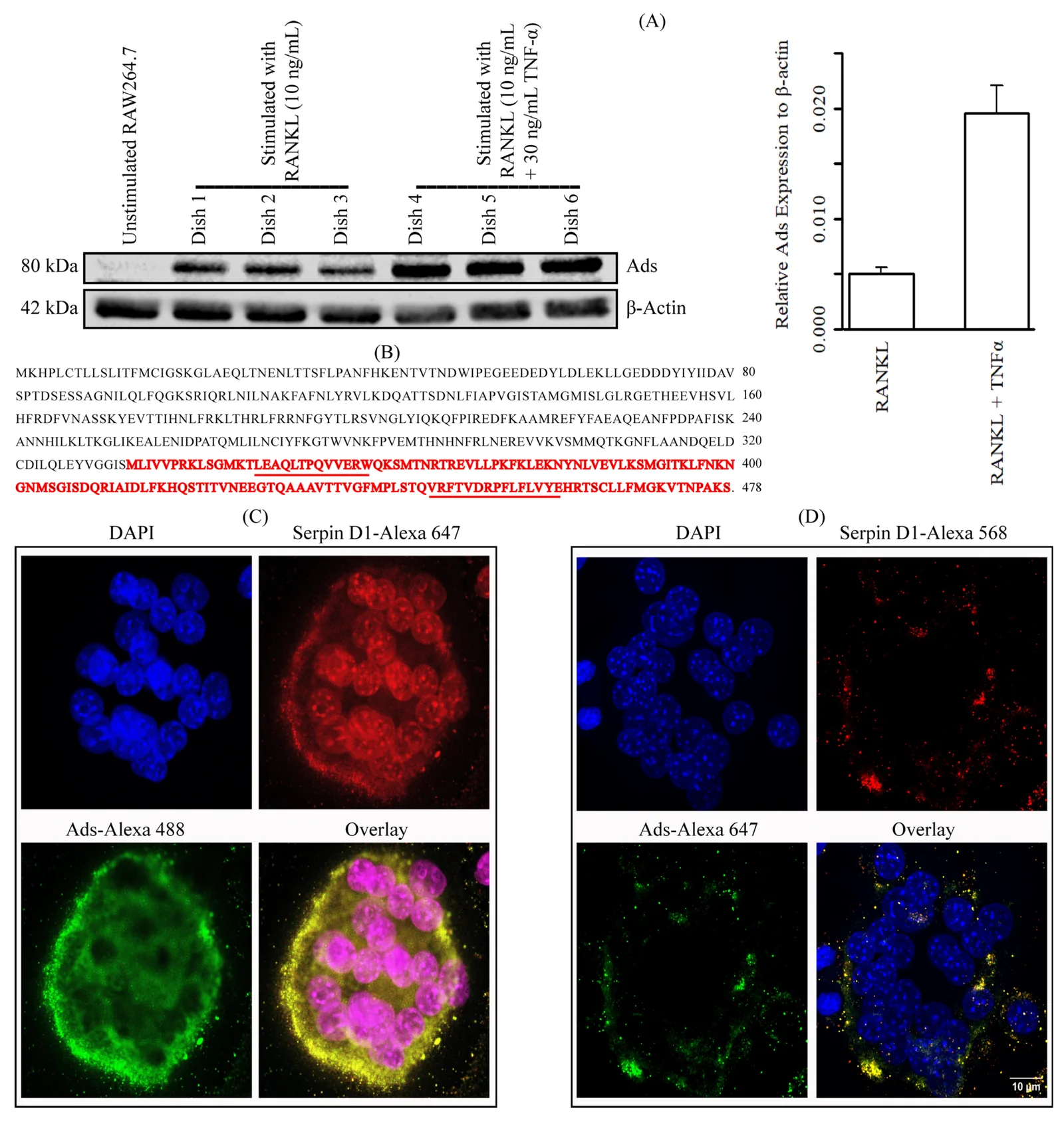
Mass spectrometry analysis identified Serpin D1 in mouse osteoclast lysates immunoprecipitated with an Ads antibody. RAW264.7 cells were stimulated with soluble RANKL (10 ng/mL) for 4 days, with or without TNF-α (30 ng/mL) addition. Gelsolin or Ads in the cell lysates was immunoprecipitated by protein A beads, in conjunction with rabbit-derived antibodies respectively against gelsolin or Ads. Normal rabbit IgG was used as the control for the rabbit polyclonal antibodies. (**A**) Western blot analysis of Ads expression in RAW264.7 cells treated with RANKL and with TNF-α stimulation. Data in panel (**A**) show one representative blot of three technical replicates. The cell treatments and Western blot analysis were performed independently four times. The difference between mean RANKL and RANKL + TNF-a was examined with Student’s *t*-test (*p* < 0.01). (**B**) Mass spectrometry analysis identified 2 unique Serpin D1 peptides (underlined; from the C-terminal) in cells treated with RANKL + TNF-α. The red color text indicates the amino acid sequence of the truncated Serpin D1. (**C**) Immunofluorescence staining showed colocalization (yellow) of Serpin D1 (red) with Ads (green) at the subcortical regions of osteoclasts derived from RAW264.7 cells. The cells were counterstained with DAPI (blue, appearing magenta in the merged image). Images of different channels and the overlay image of a representative stained cell are shown. (Objective magnification 40×). (**D**) Wild-type osteoclasts treated with RANKL (60 ng/mL, 4 days) from RAW264.7-H10 cells [10] were immunostained for Serpin D1 (red) and adseverin (green, pseudo-colored) and imaged with a Zeiss Airyscan confocal microscopy. Colocalization analysis of 10 multinucleated osteoclasts from 3 different cultures were analyzed by calculation of Pearson’s correlation coefficient. (Objective magnification 60×).

Adseverin was expressed at very low levels in untreated, parental RAW264.7 cells. When cells were treated with RANKL (10 ng/mL) to induce osteoclast formation, there were substantial increases in Ads expression (>1000-fold). In an earlier report [34], treatment of monocytes with permissive levels of RANKL and TNF-α induced osteoclastogenesis by direct stimulation of monocytes. Similarly, we found that Ads expression levels was increased by 2- to 4-fold in cells treated with RANKL + TNF-α (30 ng/mL) compared with RANKL alone (*p* < 0.05; Student’s *t*-test; Figure 1A), consistent with earlier data [29].

**Figure 2:**
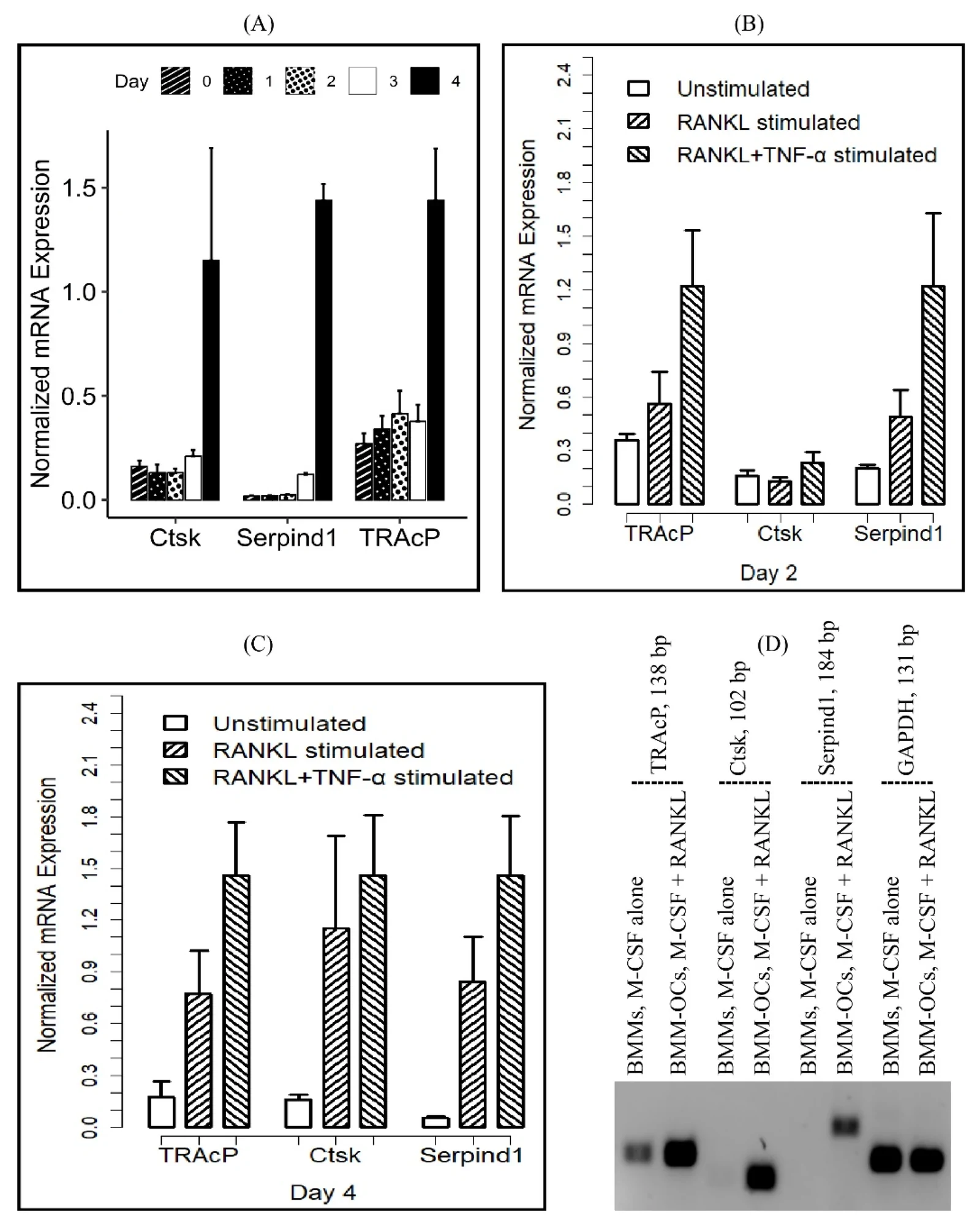
Effect of TNF-α on RANKL-induced *Serpind1* expression in osteoclasts. (**A**) RT-qPCR analysis of *Acp5* (TRAcP), *Ctsk*, and *Serpind1* expression at the indicated days of culture showed that expression of these genes was highest on day 4 (*p* < 0.01). Their expression levels were significantly increased following treatment with RANKL (10 ng/mL) compared with those in undifferentiated day 0 RAW264.7 osteoclast precursor cells. (**B**,**C**) *Acp5* (TRAcP) expression was upregulated on days 2 and 4 in stimulated cells compared with unstimulated cells, indicating that the cells responded to the stimulation and initiated osteoclast differentiation and formation. The expression levels of the three genes were compared between cells treated with RANKL alone and those treated with RANKL plus TNF-α. *Acp5* (TRAcP) expression was markedly enhanced by the addition of TNF-α in the presence of RANKL on both day 2 and day 4. An increasing trend in the expression of the other two genes was also observed following the addition of TNF-α. Data are expressed as mean ± SD from three independent experiments. Statistical significance was determined using a two-tailed Welch’s unpaired *t*-test. (*p* < 0.01; panel (**B**,**C**)) (**D**) Measurement of *Serpind1* mRNA expression in mouse primary bone marrow monocyte (BMM)-derived osteoclasts and in monocytic osteoclast precursor cells. Estimates of mRNA abundance used semi-quantitative RT-PCR. *Acp5* (TRAcP) and *Ctsk* expression were used to validate osteoclast formation. GAPDH expression was used to assess the authenticity of the reverse transcription. Primers used for *Serpind1* RT-qPCR and semi-quantitative PCR are located in mouse *Serpind1* exon 3 (forward) and 4 (reverse) [25]. The size of the amplicons is indicated above each gene.

Tandem mass spectrometry analysis of Ads immunoprecipitates was used to identify Ads-associating proteins in RAW264.7 cell-derived osteoclasts that had been induced by RANKL and TNF-α (Table 1). Of those proteins that were identified with low type 1 error (*p* < 0.01), we noted other Ads-associating proteins (e.g., Dock7, Ran) but did not pursue these as we focused on novel proteins that were likely to regulate the function of Ads. We focused on Serpin D1 because, like Ads, its expression in osteoclast precursors was induced by RANKL and was further enhanced by TNF-α (see Figure 2A–C below).

Two tryptic peptides (Figure 1B; TLEAQLTPQVVER and FTVDRPFLFLVYEHR) were mapped to the C-terminal of Serpin D1, indicating that this protein was a high probability (*p* < 0.001) Ads-associating protein. This finding is consistent with earlier data showing that RANKL induced *Nfatc1*-dependent transcripts in osteoclasts, one of which was *Serpind1* [25]. Serpin D1 was also specifically detected in pull-downs with Ads antibody from osteoclast lysates. Mass spectrometry did not detect Serpin D1 in samples immunoprecipitated with an anti-gelsolin antibody. Notably, mass spectrometry analysis of anti-Ads immunoprecipitates from osteoclast lysates detected peptides corresponding to the truncated form of Serpin D1, but not peptides uniquely corresponding to other Serpin D1 isoforms. We also looked for structural homologs of Serpin D1 such as Serpin C1 and Serpin A5 but we did not detect any peptides in the immunoprecipitates that matched the sequences of these proteins. Immunostaining showed that in osteoclasts, Serpin D1 localized to sub-cortical sites and to nuclear regions. Ads colocalized with Serpin D1 in sub-cortical regions (Pearson’s correlation coefficient r = 0.56; n = 25 cells; *p* < 0.01; Figure 1C). We conducted the same type of immunostaining experiment but with different secondary antibodies. In these analyses (Figure 1D) wild-type osteoclasts differentiated with RANKL (60 ng/mL, 4 days) from RAW264.7-H10 cells [10] were immunostained for Serpin D1 (red) and adseverin (green, pseudo-colored) and imaged with a Zeiss Airyscan confocal microscopy. Colocalization analysis of 10 multinucleated osteoclasts in each of 3 different cultures indicated a Pearson’s correlation coefficient of 0.70 ± 0.03 for Serpin D1 and Ads.

### 3.2. Regulation of Serpind1 Expression in Osteoclasts

We examined *Serpind1* mRNA expression in RAW264.7 cells treated with RANKL (10 ng/mL; 1–4 days). Cells at Day 0 (no RANKL added) were used as controls. Time-course analyses of *Serpind1* mRNA were measured by RT-qPCR, which showed that after 4 days, *Acp5* (TRAcP) and *Ctsk* expression was increased (5- and 7-fold at day 4 vs. day 0), indicating osteoclast formation in response to 4-day RANKL (10 ng/mL). There was increased *Serpind1* mRNA expression (75-fold at day 4 vs. day 0) (Figure 2A), which was contemporaneous with increased mRNA expression of the osteoclast markers, *Acp5* (TRAcP) and *Ctsk*. In separate experiments RAW264.7 cells treated with RANKL (10 ng/mL) + TNF-α (30 ng/mL) for 2 days (Figure 2B) or 4 days (Figure 2C) exhibited >2-fold increased mRNA expression of *Acp5* (TRAcP) compared with cells treated with RANKL alone. An increasing trend in *Ctsk* and *Serpind1* mRNA expression was also observed following TNF-α treatment in the presence of RANKL. As determined by semi-quantitative RT-PCR, *Serpind1* mRNA was also expressed by osteoclasts differentiated from primary mouse bone marrow monocytes after stimulation with M-CSF and RANKL but not with M-CSF alone (Figure 2D).

### 3.3. Serpind1 Isoform Expression in Osteoclasts

**Figure 3:**
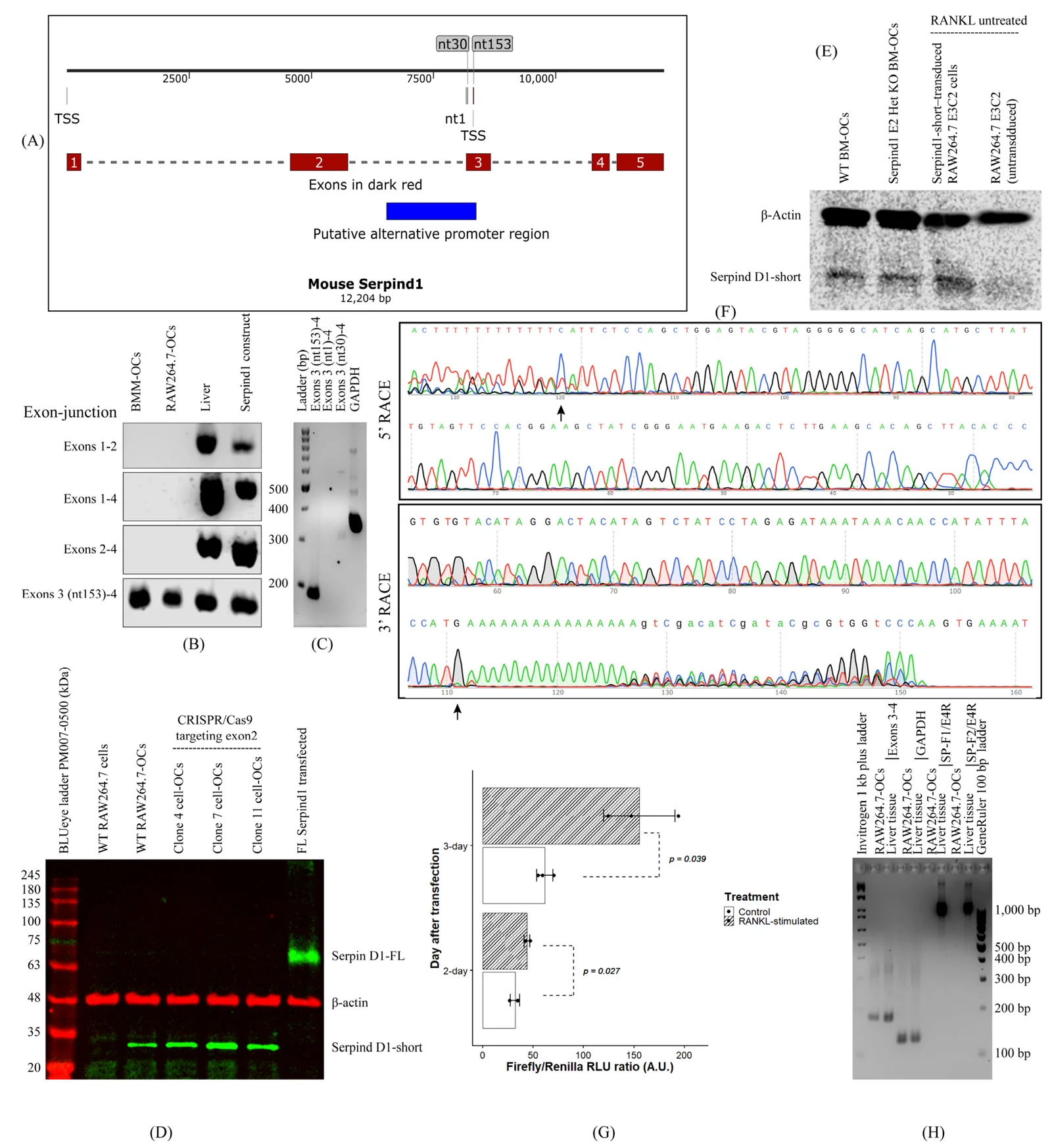
Serpin D1 in mouse osteoclasts is expressed as a truncated isoform. (**A**) Schematic representation of the mouse *Serpind1* gene, showing transcription start sites (TSSs) corresponding to the full-length and truncated transcripts; (**B**) RT-PCR was conducted with forward and reverse primers (described in the Materials and Methods) targeting groups of exons: exons 1 and 2, exons 1 and 4, exons 2 and 4, exons 3 and 4 of full-length *Serpind1*, respectively. (**C**) RT-PCR analysis of RAW264.7 cell-derived osteoclasts was conducted to examine transcription of the truncated *Serpind1*; (**D**) WT RAW264.7 cells, and *Serpind1* KO (E2) RAW264.7 cell lines (clones #4, #7 and #11) were treated with RANKL (60 ng/mL) for 4 days to induce osteoclast formation. Osteoclasts derived from WT and *Serpind1* KO (E2) cell clones were examined for protein expression of Serpin D1; (**E**) Analysis of the truncated Serpin D1 isoform in osteoclasts (OCs) derived in vitro from bone marrow (BM) cells of wild-type and *Serpind1* KO (E2) knockout mice. RAW264.7 cells with a CRISPR-mediated deletion of exon 3 (E3) Clone 2 (E3C2) were transduced with a retrovirus expressing the short Serpin D1 isoform and used as the positive control, while untransduced E3C2 cells served as the negative control; (**F**) Analysis of the *Serpind1* transcript in mouse osteoclasts assessed the start of the mRNA sequence (commences with TSS2 (5′-CATTCTCCAGCTGG…) which terminated at TTTACCATG-3′. Note that the termination is immediately followed by the addition of a poly(A) tail; (**G**) A 1996 bp upstream region and a 58 bp downstream sequence (blue bar in (**A**)) flanking TSS2 was cloned into pGL4.10[Luc2] and transfected into RAW264.7 cells. The transfected cells were treated with RANKL for two or three days and the activity of Luciferase was measured. Data are mean ± standard deviation. Statistical analysis was performed with Student’s *t*-test and *p*-values were estimated. (**H**) RT-PCR was used to analyze RNA isolated from RANKL-induced RAW264.7 osteoclasts. Mouse liver was used as a positive control for full-length *Serpind1*. Primers targeting the signal peptide region and exons 3–4 were used. These experiments were conducted to assess whether the signal peptide-coding sequence in full-length *Serpind1* was absent (sample lanes 5 and 7) in the truncated isoform.

The full-length mouse *Serpind1* gene consists of five exons and is transcribed from the first transcription start site (Figure 3A). RT-PCR showed that exons 1 and 2 were absent in osteoclasts derived from bone marrow or RAW264.7 cells. Primer pairs designed to hybridize within exons 1 and 2, or to span exon-exon junctions from exon 1 or exon 2 to exon 4, failed to amplify any products. In contrast, a primer pair with the forward primer located in exon 3 and the reverse primer located in exon 4 [25] enabled robust amplification of the transcript. The cDNA from mouse liver and a plasmid containing full-length *Serpind1* (Sino Biological; Cat. No. MG50121) served as positive controls (Figure 3B). We mapped the transcription start site of the truncated isoform with RT-PCR using primer pairs targeting different regions of exon 3. Reactions with primer sets that used forward primers located at the beginning of exon 3 nucleotide (nt) 1 or 30 together with a reverse primer in exon 4 did not yield detectable PCR products. In contrast, when a forward primer positioned at nt 153 of exon 3 was used with the same reverse primer, a clear PCR product was obtained (Figure 3C). These results indicate that the truncated transcript is generated from within exon 3, between nucleotide 30 and nucleotide 153 of the full-length exon.

We also employed CRISPR/Cas9-mediated genome editing to target the full-length *Serpind1* exon 2 in RAW264.7 cells. Sanger sequencing of genomic DNA from edited cells confirmed the deletion of 35 bp (5′-ACTCTTCTTTCTCTCATCACATTTATGTGTATTGG-3′), 25 bp (5′-ACTCTTCTTTCTCTCATCACATTTA-3′), or an 49 bp (5′-CTGCACTCTTCTTTCTCTCATCACATTTATGTGTA-3′) in exon 2 of *Serpind1* in three individual cell clones, respectively. These three individual cell clones were designated as #4, #7 and #11. WT RAW264.7 cells and the three clonally derived cell lines with a deletion in exon 2 were treated with RANKL (60 ng/mL) for four days to induce osteoclast differentiation. Western blot analysis showed no detectable Serpin D1 in unstimulated WT RAW264.7 cells. In contrast, Serpin D1 was observed in osteoclasts differentiated from WT cells and in three *Serpind1* CRISPR KO cell clones (#4, #7 and #11) in which exon 2 (E2) was deleted. The molecular mass of Serpin D1 detected in these samples was ~30 kDa (Figure 3D and Figure S1), which is smaller than full-length Serpin D1 (65 kDa). Osteoclasts derived from bone marrow cells of WT and *Serpind1* heterozygous KO (Tokushima, exon 2-targeted) mice similarly expressed only the truncated Serpin D1 isoform. Comparable protein expression levels of the truncated isoform were observed in both genotypes (Figure 3E). These data indicate that in mouse osteoclasts, *Serpind1* is transcribed as a truncated variant, which is distinct from the full-length protein. These data are consistent with RT-PCR results (Figure 3B), indicating that deletion of exon 2 does not affect the transcription or translation of the truncated *Serpind1* variant.

By 5′-RACE analysis we identified the transcription start site (Figure 3F, upper panel, arrow) within exon 3 of the full-length *Serpind1*. Based on this analysis and the molecular mass of Serpin D1 detected in osteoclasts by immunoblotting, we predicted that the translation of the truncated *Serpind1* variant in osteoclasts begins at the first ATG downstream of the transcription start site of the truncated *Serpind1* transcript. The predicted protein sequence (Figure 1B) comprises residues 334–478 (MLIVVPR…VTNPAKS), which are located within the serpin domain of full-length Serpin D1.

In addition to these experimental approaches, we used 3’-RACE and found that transcription terminates immediately before the poly(A) tail (Figure 3F, lower panel, arrow). We also assessed promoter activity associated with the intron located between exons 2 and 3 of full-length *Serpind1*. A genomic DNA fragment from RAW264.7 cells, encompassing part of the intron and a small portion of exon 3 (Figure 3A, shown as a blue bar), was amplified and cloned into a luciferase reporter. This construct was transfected into RAW264.7 cells, which were then treated with RANKL to evaluate promoter activity driven by this region. There was increased RANKL-induced promoter activity two and three days after transfection compared with untreated controls (Figure 3G).

As the truncated Serpin D1 is intracellular and not secreted, we considered that this Serpin D1 variant may lack a signal peptide. Accordingly, RT-PCR was performed using RNA isolated from RANKL-induced RAW264.7 osteoclasts. Mouse liver was examined as a positive control for full-length *Serpind1*. Primers targeting the signal peptide region and exons 3–4 were used for the analyses. The data indicated that the signal peptide-coding sequence present in full-length *Serpind1* was absent (sample lanes 5 and 7) from the truncated variant (Figure 3H).

### 3.4. Deletion of Serpind1 Exon 3 Impairs RANKL-Induced Osteoclastogenesis

**Figure 4:**
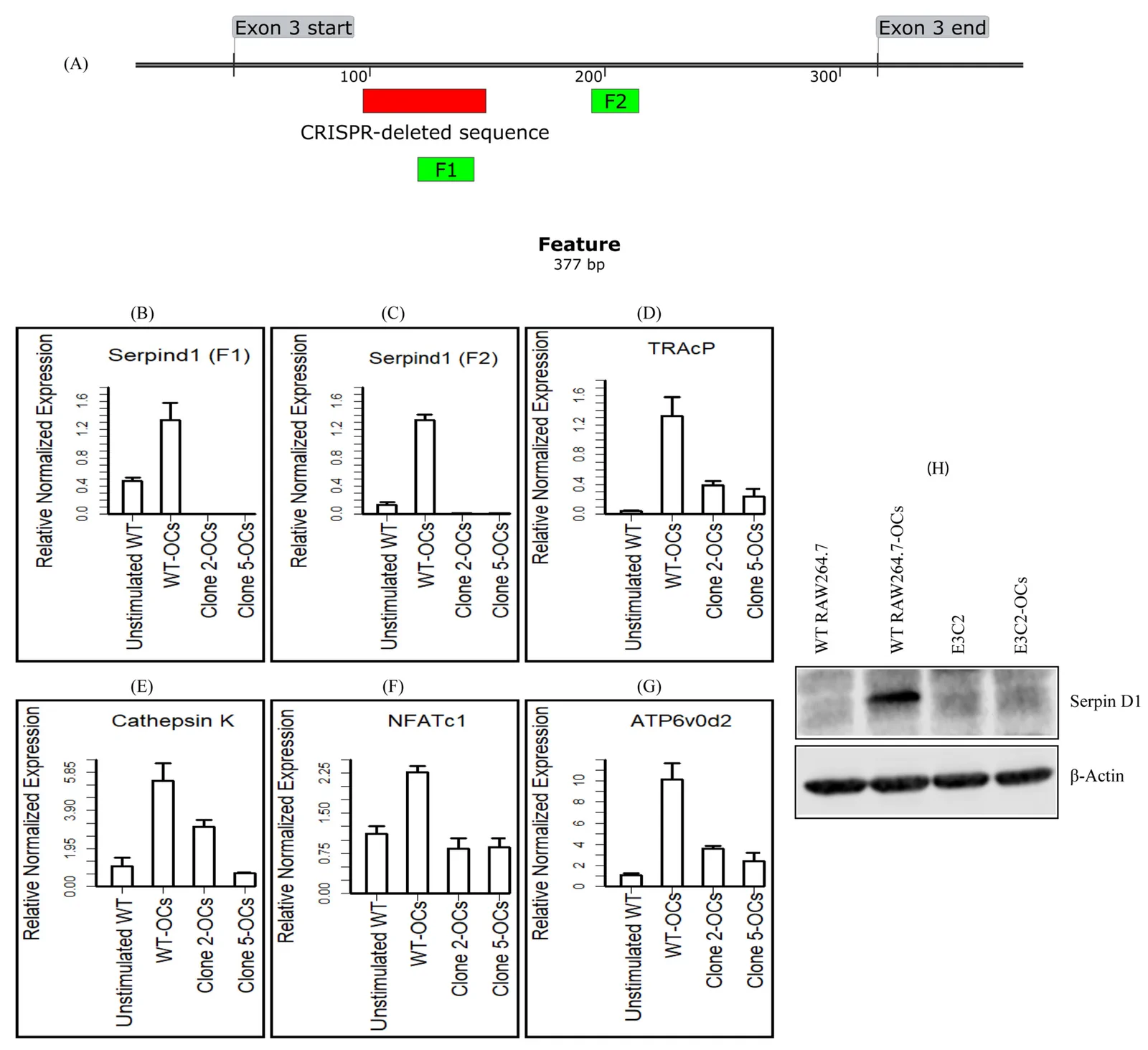
Genotypic analysis of *Serpind1* (exon 3) KO (E3KO) cells. (**A**) Genomic DNA extracted from cells as template for PCR was used to amplify the RNA guides in the targeted region. PCR products were cloned into a TA-vector for Sanger sequencing. Results show 52 bp was deleted from exon 3 of *Serpind1* in both cell lines; (**B**) Cells were stimulated with RANKL for 4 days. Unstimulated wild-type cells were used as controls. Forward primer F1. (5′-TCTCCATGATGCAAACTAAAGGGA-3′) within the deleted region and reverse primer [25] within exon 4 were used for RT-qPCR. (**C**) Deletion of *Serpind1* in *Serpind1* KO (E3) cell-derived osteoclasts was confirmed with a pair [25] of primers in which the forward primer F2 is located downstream of the deleted region; the reverse primer is located in exon 4. (**D**–**G**) Compared with osteoclasts derived from wild-type cells, osteoclasts derived from *Serpind1* exon 3-deleted cell lines 2 and 5 expressed significantly lower levels of the osteoclast marker genes *Acp5* (TRAcP) and *Ctsk,* as well as the osteoclast differentiation- and function-associated genes *Nfatc1* and *Atp6v0d2*. All gene expression data were normalized to GAPDH. Experiments were repeated 4 times on separate occasions. Statistical differences were examined with one-way ANOVA followed by Fisher’s LSD multiple-comparison test. (**B**–**D**; *p* < 0.02) (**E**–**G**; *p* < 0.05) (**H**) Western blot analysis showed truncated Serpin D1 only in osteoclasts that were differentiated from wild-type RAW264.7-H10 cells [10]. Serpin D1 was not detected in precursor cells when not treated with RANKL or in osteoclasts derived from *Serpind1* KO E3 cells. Data are representative results from one set of replicated experiments and are mean ± SEM of triplicate measurements.

As the deletion of exon 2 did not affect *Serpind1* expression in osteoclasts (Figure 3D,E), CRISPR/Cas9-mediated genome editing was used to delete a portion of *Serpind1* exon 3, which contains the alternative transcription start site required for expression of the truncated variant. Two independent, clonal RAW264.7 cell lines, designated #2 (E3C2) and #5 (E3C5), were generated by standard limiting dilution. Both clones harbored the identical deletion of the following sequence:

GAATGAGAGAGAAGTAGTCAAAGTCTCCATGATGCAAACTAAAGGGAATTTC (Figure 4A). Given the heterogeneous nature of RAW264.7 cells, the two independent clones with the same deletion were used in subsequent experiments to confirm that the observed effects were due to the gene knockout rather than clone-specific differences. Analysis of *Serpind1* mRNA by RT-qPCR showed no *Serpind1* expression in the *Serpind1* KO (E3) cell-derived osteoclasts (Figure 4B,C). Deletion of *Serpind1* exon 3 significantly reduced the expression of *Acp5* (TRAcP), *Ctsk*, *Nfatc1*, and *Atp6v0d2* in osteoclasts compared with those generated from WT cells (Figure 4D–G). Western blot analysis using an anti-Serpin D1 antibody (R&D Systems, AF1265) and IRDye^®^ 680RD Donkey anti-Goat IgG, P/N 926-68074 secondary antibody confirmed that the truncated Serpin D1 was expressed only in osteoclasts derived from wild-type RAW264.7 cells (Figure 4H). No Serpin D1 expression was detected in precursor cells (RANKL-untreated RAW264.7 cells), including both wild-type and *Serpind1*-KO (E3) cells. Serpin D1 expression was also absent in osteoclasts derived from the *Serpind1* (E3) CRISPR knockout cells.

### 3.5. Interaction of Purified Serpin D1 with Ads

**Figure 5:**
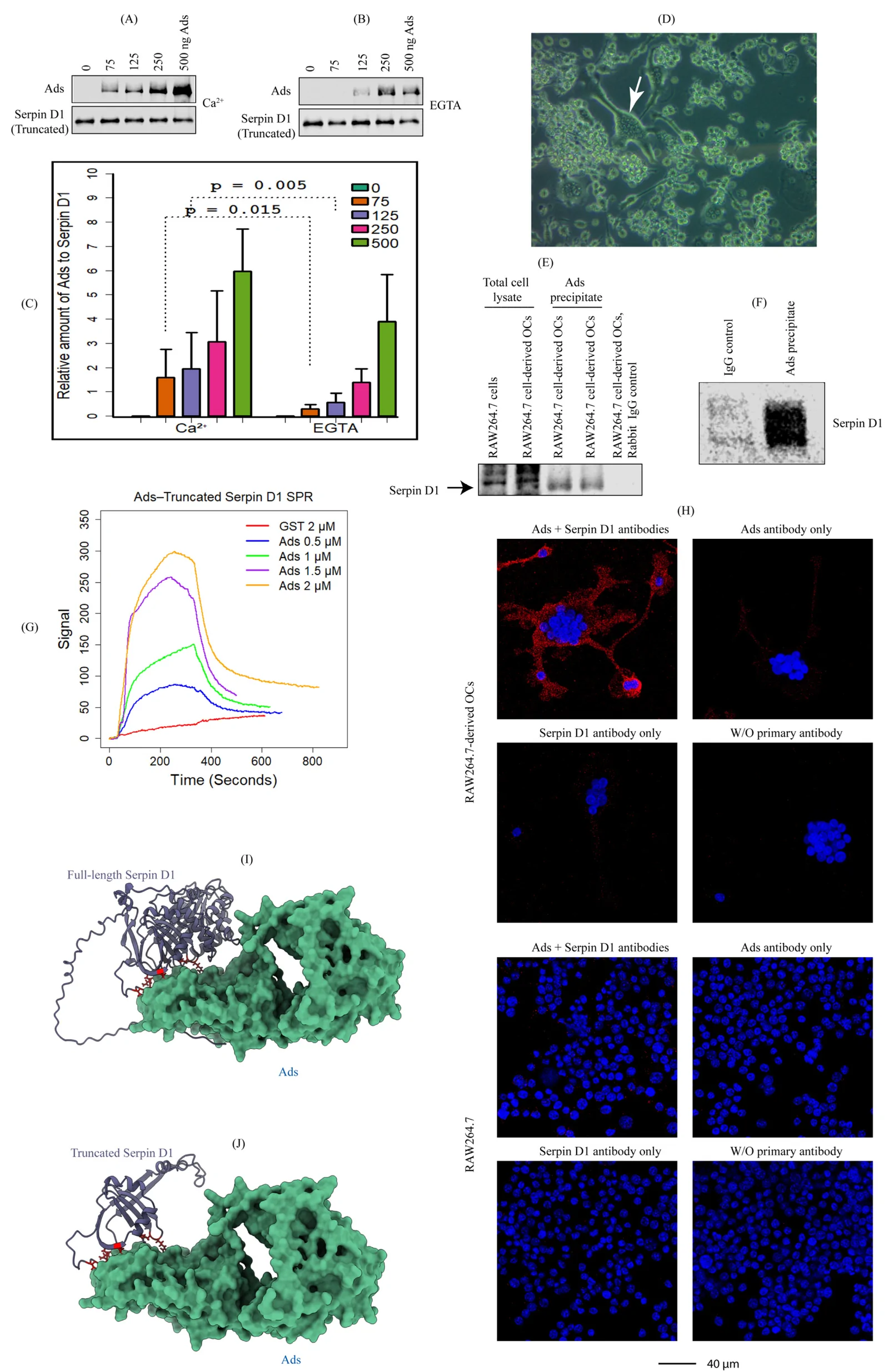
Pull-down assay, immunoprecipitation, SPR, and PLA analyses confirmed Serpin D1 and Ads interaction. Glutathione Sepharose 4b-Serpin D1 (truncated) was used to pull down recombinant Ads in a protein-protein interaction buffer containing either Ca^2+^ (**A**) or EGTA (**B**). Results show that Ads and Serpin D1 interact, and that the interaction between Ads and Serpin D1 was Ca^2+^ dependent (**C**, quantification). These experiments were performed independently three times. Data are presented as mean ± standard error of means. Statistical analysis was performed using Student’s *t*-test for estimation of *p*-values. (**D**) RAW264.7 cells were stimulated with RANKL (60 ng/mL) plus TNF-α (30 ng/mL) to induce osteoclast formation (see pointed arrow). (**E**) Cells that were treated as described in panel (**D**) were lysed in RIPA buffer containing protease inhibitors and CaCl_2_ (1 mM). Immunoprecipitation was performed with anti-Ads antibody. Serpin D1 (truncated isoform) was detected in the Ads precipitates but not in the precipitates from the isotype control. (**F**) RAW264.7 cells transfected with the truncated isoform of *Serpind1* differentiated into osteoclasts upon exposure to RANKL. Immunoprecipitates of Ads revealed the presence of Serpin D1. Surface Plasmon Resonance-SPR (**G**), Proximity Ligation Analysis-PLA (**H**), and computer modeling (**I**,**J**) further support an interaction between Serpin D1 and Ads. For quantification of PLA, the proportion of signal (number of red dots) that overlaid cell areas and the number of red dots that overlaid equivalent areas on the slide but without cells was counted for 50 cells.

As cytoplasmic [Ca^2+^] signaling is increased during RANKL-mediated osteoclastogenesis [43], we examined whether the interaction of Serpin D1 with Ads in vitro was dependent on Ca^2+^. First, we measured the size of fused cells after treatment with the intracellular Ca^2+^ chelator, BAPTA/AM. The fused cells were reduced in size by 10-fold (Supplementary Figure S2), suggesting that cell fusion was dependent in part on Ca^2+^ signaling. Arising from this observation, we conducted experiments to evaluate whether the association of Serpin D1 with Ads was Ca^2+^ dependent. Glutathione S-transferase-tagged recombinant Ads was expressed in *E. col*i BL21 cells, bound to glutathione Sepharose 4B resin and incubated with recombinant, full-length Serpin D1 at various concentrations in a buffer containing 200 µM Ca^2+^. When the concentration of Ads bound to resin was kept constant, qualitative analysis showed that the amount of Serpin D1 subsequently eluted from the bound Ads was related to the concentration of the Serpin D1 in the incubation reaction (Figure 5A). The impact of Ca^2+^ on the binding of Serpin D1 to Ads in vitro was assessed in buffers containing Ca^2+^ (Figure 5B) or EGTA (Figure 5C). These experiments indicated that the binding of Serpin D1 to Ads is Ca^2+^-dependent. Analysis of replicate samples (n = 3) showed that compared with controls (buffer containing 200 µM Ca^2+^), there was 55% (*p* < 0.05) less Serpin D1 binding to Ads in reactions conducted in a buffer containing 1 mM EGTA, a Ca^2+^ chelator. The amount of binding of Serpin D1 to Glutathione S-transferase in the absence of Ads (as a control) was very small (Supplementary Figure S3).

We examined whether truncated Serpin D1 and Ads associated with one another in RAW264.7 cells that had been induced to form osteoclasts by treatment with RANKL and TNF-α. Osteoclast formation was confirmed following RANKL and TNF-α treatment (Figure 5D, white arrow). Truncated Serpin D1 was not detected in untreated RAW264.7 cells (Figure 5E, lane 1) but was detected in RAW264.7 cell-derived osteoclasts (lane 2). Truncated Serpin D1 was also detected in cell lysates immunoprecipitated with anti-Ads antibody (lanes 3 and 4). The ratio of the blot densities of Serpin D1 pulled down in Ads immunoprecipitates compared to isotype control antibody in 4 analyses was 12.5 ± 1.3 (mean ± standard deviation).

The association between Serpin D1 and Ads was also examined by transfecting RAW264.7 cells with a truncated Serpin D1 expression construct (Figure 5F). This construct was generated by site-directed mutagenesis to remove the N-terminal region (nucleotides 1-855), which is not expressed in osteoclasts. Transfected cells were treated with RANKL for 2 days to induce Ads expression. Ads immunoprecipitates prepared from these cells contained the truncated Serpin D1 while samples incubated with normal rabbit IgG did not.

The interaction between Serpin D1 and Ads was also assessed by surface plasmon resonance (SPR) analysis of purified proteins and by PLA analysis of intact osteoclasts. SPR analysis demonstrated binding of truncated Ads and Serpin D1, which was manifest in a Serpin D1 dose-dependent manner (Figure 5G). PLA analyses of RAW264.7-derived osteoclasts (treated with RANKL; top panels, Figure 5H) and untreated RAW264.7 cells (lower panels, Figure 5H) showed that with the same image acquisition parameters, the PLA signal (colored red, indicative of Ads-Serpin D1 interaction) was seen only in osteoclasts. Samples incubated with a single primary antibody or with no primary antibody served as controls and showed no red (PLA) signal. For 3 different preparations, we computed the proportion of signal (number of red dots) that overlaid cell areas compared with the number of red dots that overlaid equivalent areas on the slide but without cells. The proportion for the Ads plus Serpin D1 antibodies group was 17.3 ± 1.7 (mean ± standard deviation) and for the Serpin D1 antibody only group was 1.2 ± 0.9 (*p* < 0.01).

We used computational modeling methods (Alpha Fold 3 and molecular docking models) to examine potential interacting domains of Serpin D1 and Ads. The top-ranking model showed docking configurations of both full-length and truncated Serpin D1 (receptor) with Ads (ligand). Four contact points located in exon 3 to exon 5 of *Serpind1* (amino acids 334–478) were identified. (Figure 5I,J, Supplementary Videos S1 and S2). Detailed amino acid residue and atomic-level contact data are provided in Supplementary Tables S2 and S3. Individual contact residues between truncated Serpin D1 and Ads are highlighted in red and these data are summarized in Table 2.

### 3.6. Serpin D1 Affects Ads-Mediated Regulation of Subcortical Actin Filament Assembly

**Figure 6:**
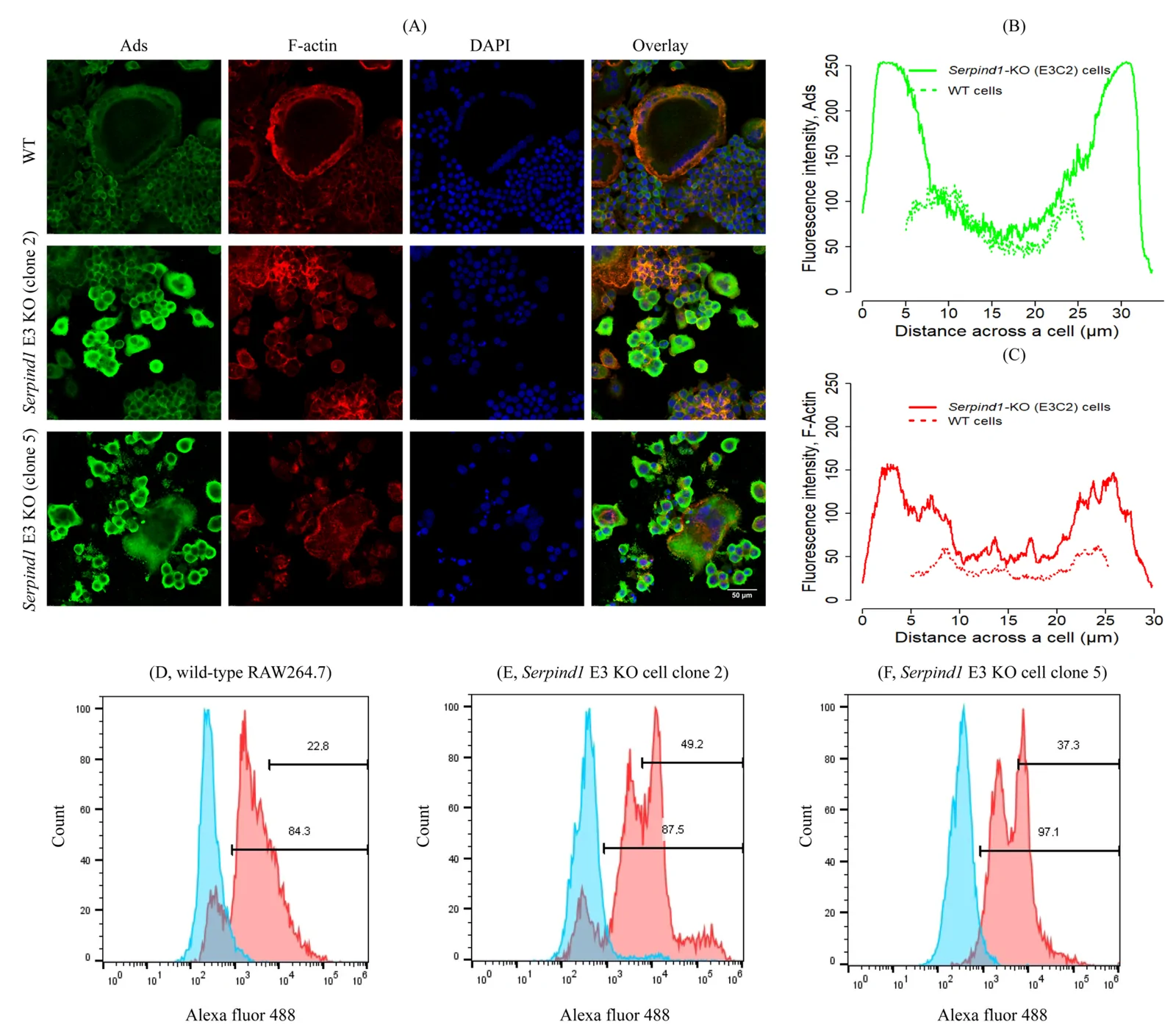
Increased Ads expression in WT vs. *Serpind1*-KO cells treated with RANKL. WT and *Serpind1*-KO (E3) cells (clones 2 and 5) were stimulated with RANKL (60 ng/mL) for osteoclast formation. Immunofluorescence staining showed more abundant Ads in *Serpind1*-KO pre-fusion osteoclasts/osteoclasts than WT (**A**). Representative plots show the measurement of fluorescence intensity of Ads (**B**) and actin filaments (**C**) in subcortical regions by measuring fluorescence intensity along a line across the middle of the cell. Three-dimensional reconstructions of the confocal microscopy optical sections are shown as videos in the supplemental data (Supplementary Videos; WT culture and exon 3 deletion by CRISPR). For the (**B**,**C**) panels, measurements were performed on 12 cells from each genotype. Representative images from the wild-type and *Serpind1*-KO (E3) cell line clone 2 (E3C2) are shown. Data obtained by immunostaining was confirmed by flow cytometry. Although there was no difference between the wild-type (**D**) and *Serpind1*-KO cells (**E**,**F**) relating to the total number of Ads positive (lower gate), the proportion of the intensively Ads-stained *Serpind1*-KO cells was twice that of the wild-type cells (upper gate). For panels (**D**–**F**) that show flow cytometry frequency distribution data, analyses were performed on 3 separate cultures. Representative results are shown from one culture. Turquoise and red data plot cell populations depict cells stained with anti-Ads antibody that are either above threshold staining levels (red) or below threshold staining levels. The smaller, dark blue data plots depict the corresponding negative control cells that were incubated with fluorophore-conjugated secondary antibody alone and that were used to estimate positive staining thresholds.

Based on the data up to this point, we hypothesized that Serpin D1 may affect the ability of Ads to regulate the assembly of subcortical actin filaments. Accordingly, WT RAW264.7 cells and *Serpind1*-KO (E3) were treated with RANKL for 4 days. Cells were immunostained for Ads, stained with rhodamine phalloidin for actin filaments and examined by confocal microscopy (Figure 6A). Compared with WT osteoclasts, *Serpind1*-KO (E3) cells treated with RANKL showed increased abundance of Ads (>2- fold greater; *p* < 0.05; 15 independent samples; Figure 6B) in the sub-cortex as well as increased abundance of actin filaments (1.8-fold greater) (Figure 6C). Evidently, in the absence of Serpin D1 and despite high levels of Ads in the subcortex, actin filaments were more abundant than in WT cells, suggesting that the actin severing activity of Ads was reduced.

Notably, prior to fusion, *Serpind1*-KO (E3) cells were 50% wider than WT RAW264.7 cells (Figure 6A–C, cell diameters: 27.5 ± 3.7 µm vs. ~11.9 ± 1.4 µm; *p* < 0.01; n =15 randomly selected cells each from clone 5 and WT populations). Despite their failure to fuse, there was an increase in cell diameter of *Serpind1*-KO (E3) cells, compared with WT cells, suggesting that Serpin D1 restrains cortical actin remodeling during early osteoclast differentiation.

Imaging by confocal microscopy and optical sectioning (Supplementary Videos S3–S8) provided more detailed analysis of the abundance of Ads and actin filaments, as well as the effect of *Serpind1* deletion on the formation of multinucleated cells. The data obtained by immunostaining were consistent with flow cytometry analyses, indicating that the proportion of Ads positive-stained cells was two-fold higher in *Serpind1*-KO (E3) osteoclasts than WT cells (Figure 6D–F).

### 3.7. RANKL Treatment Enhances Ca^2+^ Signaling in Wild-Type but Not Serpind1-KO E3 Cells

**Figure 7:**
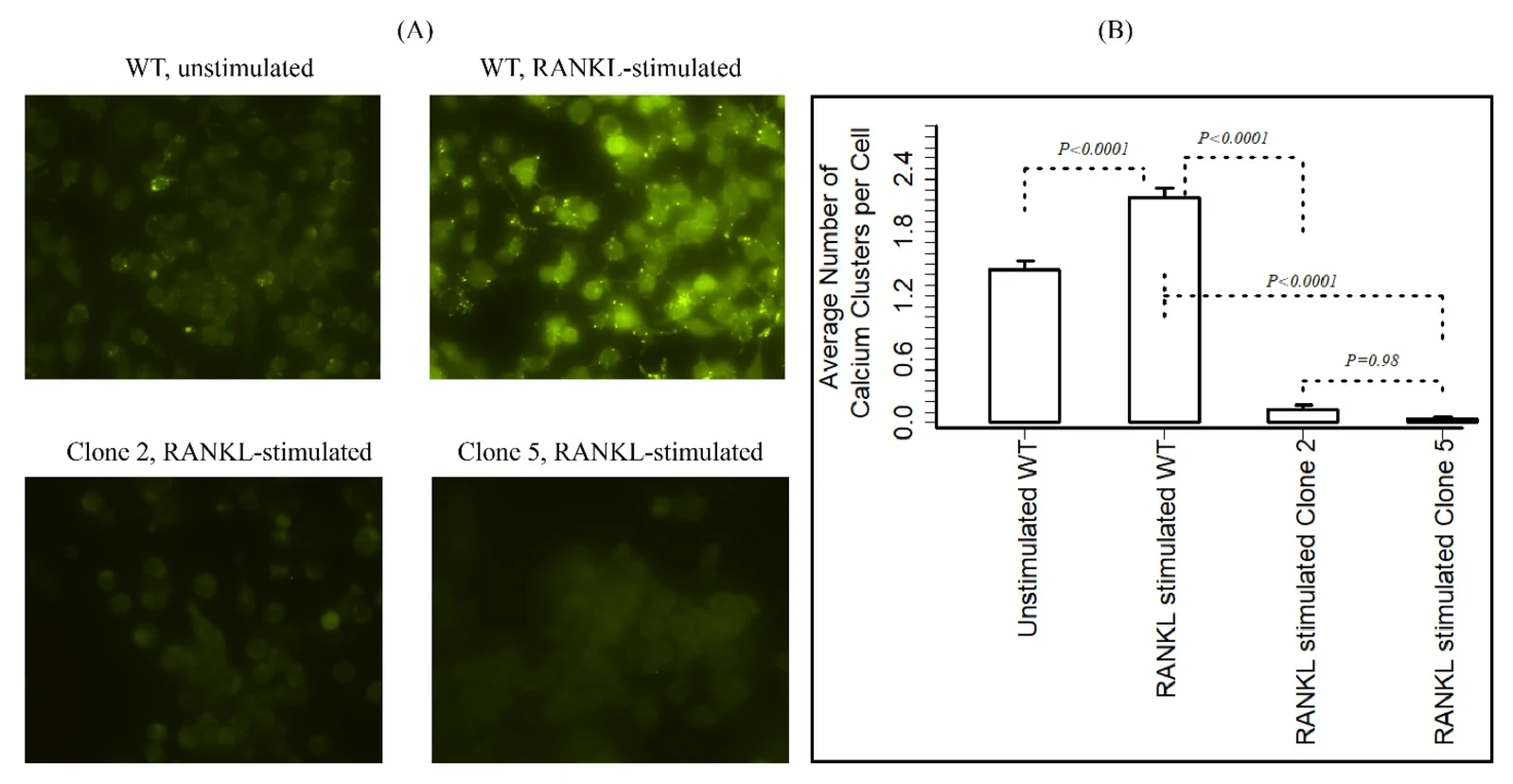
Calcium imaging shows intracellular Ca^2+^ signaling was altered in *Serpind1*-KO cells treated with RANKL. Cells were stimulated with RANKL (10 ng/mL) for two days and then RANKL-starved overnight. Calcium imaging was conducted using a 40× objective lens to image cells that were incubated in fura-2/AM followed by a second brief exposure to RANKL (**A**). Cell-associated ratiometric images of fura-2-loaded cells; (**B**) analysis of calcium clusters within cells. Total number of cells analyzed in 4 different experiments: unstimulated WT cells (n = 634); RANKL-stimulated WT cells (n = 789); RANKL-stimulated *Serpind1* KO (E3) cell lines- Clone 2 (n = 80) and Clone 5 (n = 262). Data are mean ± standard deviation from 4 separate cultures. Statistical differences (indicated in Figure 6B above histogram bars) were determined with one-way ANOVA followed by Tukey’s *post-hoc* test for individual differences.

Since Serpin D1 physically interacts with Ads, we determined whether loss of Serpin D1 alters the localization or conformation of Ads and potentially affects its ability to respond to Ca^2+^ and mediate Ca^2+^-dependent actin filament severing. Fura-2-loaded cells were examined by ratio fluorimetry to estimate intracellular Ca^2+^. Osteoclasts were generated from RAW264.7 cells by treatment with RANKL and then deprived of RANKL overnight. The next day, WT osteoclasts treated with RANKL for 10 minutes exhibited increased cytoplasmic Fura-2 fluorescence compared with untreated WT cells (Figure 7A). RANKL-treated cells also exhibited localized, small diameter (2–3 mm) circular clusters of increased [Ca^2+^]_i_ that were manifest within 10 min of adding RANKL; these Ca^2+^ clusters disappeared after ~30 min. The clusters with increased [Ca^2+^]_i_ were not endocytic vesicles containing internalized fura-2 as the clusters disappeared after membrane depolarization with KCl. The average number of Ca^2+^ clusters per cell was 30% higher in RANKL-treated WT cells (*p* < 0.01; n = 789 total cells evaluated) compared with untreated WT cells (n = 634 total cells evaluated) (Figure 7B). Increased fura-2 brightness (i.e., increased [Ca^2+^]_i_) and greatly elevated numbers of Ca^2+^ loci (clusters) following this acute RANKL treatment were manifest in WT cells but not in *Serpind1*-KO (E3) (Clones 2 and 5).

### 3.8. Serpin D1 Enhances Actin-Severing Activity of Ads

**Figure 8:**
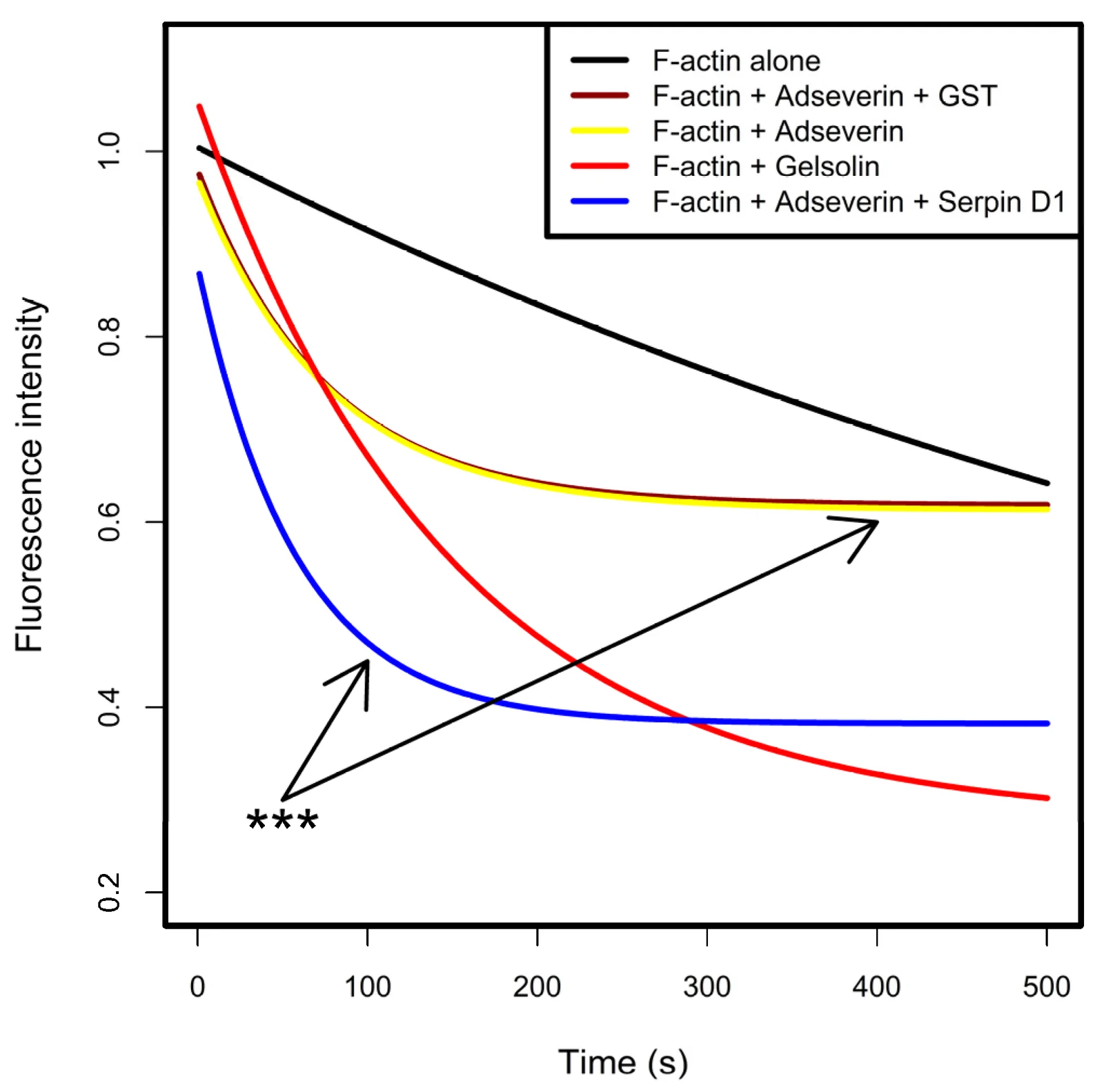
Regulation of Ads severing activity of actin filaments by Serpin D1. Data show estimated actin filament–severing activity of Ads calculated from the rate constants, which in turn were derived from fluorimetry data of pyrene (fluorescent) actin filaments conducted with different preparations as shown in the legend. F-actin + Gelsolin served as a positive control. The extent of severing was calculated as (initial fluorescence − fluorescence at 5 s after reagent addition)/initial fluorescence. Experiments were conducted three times using different preparations. Rate constants were calculated with the R program. and F-actin plus Ads and or Serpin D1 were compared from three independent experiments (ANOVA followed by Tukey’s test). F-actin plus Ads plus Serpin D1 were statistically different (*** in graph, indicates *p* < 0.001).

As RANKL treatment increased Ads expression and accumulation of subcortical actin filaments in *Serpind1*-KO (E3) cells compared with WT cells, we considered that Serpin D1 enables Ads to sever actin filaments more efficiently. Accordingly, we incubated truncated Serpin D1 with Ads in pyrene actin filament severing assays. Compared with Ads alone, truncated Serpin D1 (aa 286-478) enhanced the actin severing activity of Ads by ~55%. Representative data, which are shown as the computed rate constants estimated from 3 trials from one of 3 separate experiments are shown in Figure 8 below. Comparison of the actin severing data was conducted from estimates of the rate constants of individual treatment groups. The rate constant for F-actin plus Ads was different (*p* < 0.01; ANOVA) from the rate constants for F-actin plus Ads plus Serpin D1. The rate constant of the F-actin only sample was compared with F-actin plus Serpin D1. In 3 replicate experiments the difference between the rate constants was not statistically significant (*p* > 0.2; Student’s *t*-test for comparison of 2 different rate constants).

We also conducted time course experiments of actin severing (Supplementary Figure S4) in which we found a 2- to 3-fold increase of the rate of change of pyrene actin fluorescence in Serpin D1 + Adseverin compared with Adseverin alone.

### 3.9. Deletion of Serpind1 Exon 3 in RAW264.7 Cells Impairs RANKL-Induced Osteoclastogenesis

**Figure 9:**
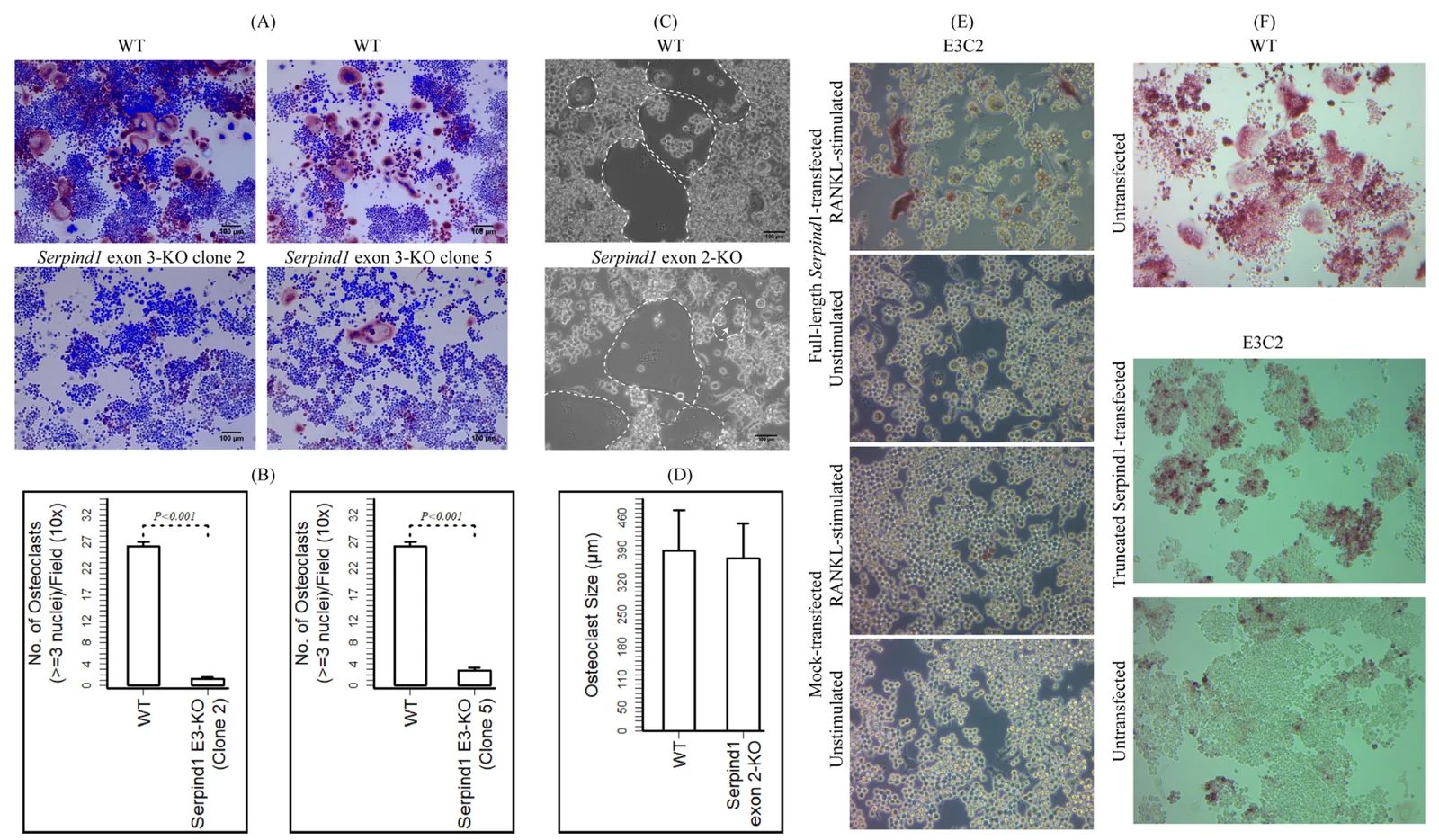
Impaired osteoclastogenesis in *Serpind1*-KO (E3KO) cells. Cells cultured in 8-well chamber slides were stimulated with RANKL for 4 days, fixed and stained for TRAcP (**A**). Quantification of TRAcP-stained cells shows *Serpind1*-KO cells with reduced capacity for osteoclast formation (**B**). Data are mean ± standard deviation from 4 separate cultures. Statistical differences were determined with Student’s *t*-test. In panel (**C**), osteoclast formation from *Serpind1* exon 2 E2KO deleted RAW264.7 cells was investigated. Representative microscopic images are shown. (**D**) Quantification of data from panel (**C**) shows osteoclasts (dash line circled) formed from the full-length *Serpind1* “exon 2” deleted cells exhibited similar size (*p*-value: >0.5) as wild-type cells, indicating that the lack of exon2 did not affect cell fusion in osteoclasts. For conduct of these experiments, ten randomly selected microscopic fields (10× objective lens) were counted to quantify osteoclast formation in (**B**), and 30 multinucleated osteoclasts (≥3 nuclei) were measured in (**D**). Data are mean ± standard errors of mean in (**B**) and mean ± standard deviation in (**D**). Statistical analysis was performed using Student’s *t*-test for comparison of two groups. (**E**). Transient transfection of a C-terminal, FLAG tagged Serpin D1 expression plasmid into E3C2 (*Serpind1* exon 3 CRISPR knockout) cells increased the proportions of RANKL-induced TRAcP-positive osteoclast formation by 8-fold compared with mock-transfected controls (*p* < 0.01; n = 4 cultures). (**F**) Stable retroviral transduction of truncated *Serpind1* into *Serpind1* CRISPR-knockout clone 2 cells produced a comparable increase in the number of RANKL-induced TRAcP-positive osteoclasts relative to untransfected controls.

Osteoclastogenesis was compared in WT and *Serpind1*-KO (E3) cell clones. As described above for the *Serpind1* KO (E3) cells (clones 2 and 5) treated with RANKL, there was no increased [Ca^2+^]_i_ and there were no bright [Ca^2+^]_i_ clusters (Figure 6B). This altered intracellular Ca^2+^ signaling in *Serpind1* KO (E3) cells suggested that Serpin D1 is important for normal Ca^2+^ signaling in response to RANKL. Since Ads-mediated actin filament severing depends on elevated Ca^2+^, our finding of reduced Ca^2+^ signaling likely diminishes Ads activity, contributing to impaired cytoskeletal remodeling and reduced osteoclastogenesis observed in *Serpind1* KO (E3) cells (Figure 9A). Osteoclast formation was reduced by 95% and by 89%, respectively, in cell clones #2 and #5 with the exon 3 deletion (Figure 9B). In contrast, deletion of exon 2 did not change osteoclast formation (Figure 9C; quantified in Figure 9D). To assess the effects of Serpin D1 re-expression, E3C2 cells were transiently transfected with a commercially available full-length *Serpind1* construct (Sino Biological, Cat. No. MG50121-CF). In parallel, GP293 packaging cells were co-transfected with pLNCX2-N1–truncated *Serpind1* and pVSV-G. Retroviral particles released into the culture supernatant were collected and used to stably transduce E3C2 cells, as described in the Materials and Methods. Compared with mock-transfected E3C2 cells (electroporated in the absence of *Serpind1* cDNA), cells expressing full-length *Serpind1* showed an approximately eightfold increase in the frequency of TRAcP-positive cells (Figure 9E). Stable expression of truncated *Serpind1* produced a comparable approximately eightfold increase of TRAcP-positive cells relative to uninfected E3C2 cells (Figure 9F).

### 3.10. Exon 2-Targeted Serpind1 Knockout

We characterized the Tokushima *Serpind1*-KO (E2) mouse model by genomic targeting. We employed PCR-based genotyping, which distinguished WT and heterozygous *Serpind1* KO mice using a diagnostic band spanning the neomycin cassette (Supplementary Figure S5A). RT-qPCR analysis of liver tissues showed a marked reduction in *Serpind1* mRNA expression in the heterozygous *Serpind1* knockout E2 mice (Supplementary Figure S5B). This finding was consistent with decreased Serpin D1 protein levels as assessed by Western blotting (Supplementary Figure S5C). As shown by TRAcP and toluidine blue staining. RANKL-induced osteoclast differentiation and resorptive activity were comparable in WT and heterozygous *Serpind1*-deficient osteoclasts cultured on either glass or dentin substrates, (Supplementary Figure S5D). As assessed by RT-qPCR, *Serpind1* expression in osteoclasts derived from the same *Serpind1* knockout E2 mice was not altered by exon 2 deletion (Supplementary Figure S5E). Consistent with these findings, expression levels of osteoclast-specific marker genes, including *Acp5* (TRAcP) and *Ctsk*, were similar between genotypes (Supplementary Figure S5E). Histological examination of distal tibial sections showed multinucleated TRAcP-positive osteoclasts on trabecular bone surfaces in both WT and heterozygous *Serpind1* KO mice, indicating that *Serpind1* deficiency does not impair osteoclast formation in vivo. Micro-CT analysis data (Supplementary Table S4) showed that for all parameters analyzed, there were no significant differences (*p* > 0.6) between WT and *Serpind1* heterozygous KO mice, except that cortical bone mineral density in the heterozygous *Serpind1* KO mice tended to be lower (*p* = 0.058).

In an effort to assess whether a specific deletion of the truncated *Serpind1* isoform could be created in C57 Bl/6 mice and thereby enable study of the role of this Serpin D1 isoform in osteoclasts and the resultant bone phenotypes in vivo, we generated LoxP-inserted mice in which exon 3 of *Serpind1* was floxed. Among the 76 pups that were born, 27 were identified as wild type and 49 as heterozygous, but no homozygous LoxP mice were obtained. Sequencing confirmed that homozygous LoxP insertion is embryonic lethal, which prevented the generation of the desired conditional knockout model. Representative genotyping results for 32 pups from heterozygous breeding using the F4/R6 primer pair (amplicon size mutant 202 bp, wild-type 134 bp) are shown in Supplementary Figure S6.

## 4. Discussion

Our main findings are that in osteoclasts, an intracellular, truncated Serpin D1 variant binds to Ads and regulates the actin severing activity of Ads, which facilitates cell fusion in Ads-dependent osteoclastogenesis. The interaction between intracellular Serpin D1 and Ads contributes to the assembly of sub-cortical actin arrays, which enables pre-osteoclast cell fusion [15]. This process may be important for the regulation of osteoclastogenesis at inflamed sites [21]. The finding of an intracellular, truncated Serpin D1 that regulates actin severing differentiates this new finding from the extracellular, full-length Serpin D1 that is involved in vascular biology.

*Serpind1* (also known as heparin cofactor II) belongs to the serpin gene superfamily and is known as an extracellular protein that plays a critical role in inflammation, immune function, tumorigenesis, cancer metastasis (reviewed in [44]) and vascular homeostasis (reviewed in [23]). Ads is an intracellular, Ca^2+^-dependent, actin severing and capping protein [45] that plays a critical role in osteoclastogenesis [10]. The actin severing activity of Ads is important for cell fusion in osteoclastogenesis because of the central role of subcortical actin in the apposition and fusion of plasma membranes of approximating pre-osteoclasts. Currently, the intracellular regulation of the severing activity of Ads is undefined. We found that in osteoclasts, deletion of *Serpind1* by CRISPR/Cas9 increased the density of subcortical actin, suggesting an intracellular role for the newly discovered, truncated Serpin D1 as a regulator of Ads function. This notion is in line with our current data showing that purified Serpin D1 promotes Ads-mediated severing of actin filaments in vitro.

Earlier studies of critical factors for osteoclast differentiation identified two sets of Nfatc1-regulated genes in mouse osteoclast precursor cells [24]. One of these proteins was Serpin D1, which is expressed in cells treated with RANKL [25]. These data are similar to our current findings and with our earlier microarray data (unpublished) in which *Serpind1* was identified in RAW264.7 cells treated with RANKL. Examination of normal bone in young mice showed that *Serpind1*-deficient mice with deletion of exon 2 are not substantially different from WT mice [25], indicating that full-length Serpin D1 is not required for osteoclastogenesis in bone development in healthy mice. However, we showed here that osteoclasts express an intracellular, truncated variant of *Serpind1* that bypassed the exon 2 deletion in these mice, suggesting the full skeletal impact from Serpin D1 deficiency might not have been fully captured in this mouse model.

As Ads is involved in osteoclastogenesis in inflammatory processes [19], we examined pre-osteoclasts treated with the pro-inflammatory cytokine TNF-α. The rationale for examining cells treated with this pro-inflammatory cytokine is that it promotes Ads expression and is an important driver of bone loss in the high prevalence of inflammatory bone diseases, including rheumatoid arthritis and periodontitis. Future investigation could be productively directed towards whether inflammatory lesions are linked to Serpin D1-enhanced bone loss. In accordance with our finding that the functionally relevant *Serpind1* transcript in osteoclasts is a splice variant lacking exons 1 and 2, its expression was not affected in *Serpind1* KO (E2) mice [25]. Microarray data show that *Serpinb1a*, *Serpinb2*, *Serpind1*, *Serpine2* and *Serpinh1* are also expressed in osteoclasts in a Nfatc1-dependent manner [25]. Conceivably, the expression of these proteins could compensate for full-length *Serpind1* deficiency in genetically altered mice.

Ads expression is strongly enhanced by TNF-α [29]. Consequently, the impact of Ads on osteoclastogenesis is mainly evident at inflamed sites [19]. Accordingly, we treated RAW264.7 cells with RANKL at low dosage (10 ng/mL) to initiate osteoclast differentiation and then added TNF-α to increase Ads expression. In mass spectrometry analysis of Ads immunoprecipitates, Serpin D1 was detected only in cells treated with RANKL plus TNF-α, but not in cells treated with low dose RANKL (10 ng/mL) alone. This finding likely reflects the lower abundance of Serpin D1-Ads complexes in cells treated only with RANKL, which may fall below the detection threshold of mass spectrometry.

**Figure 10:**
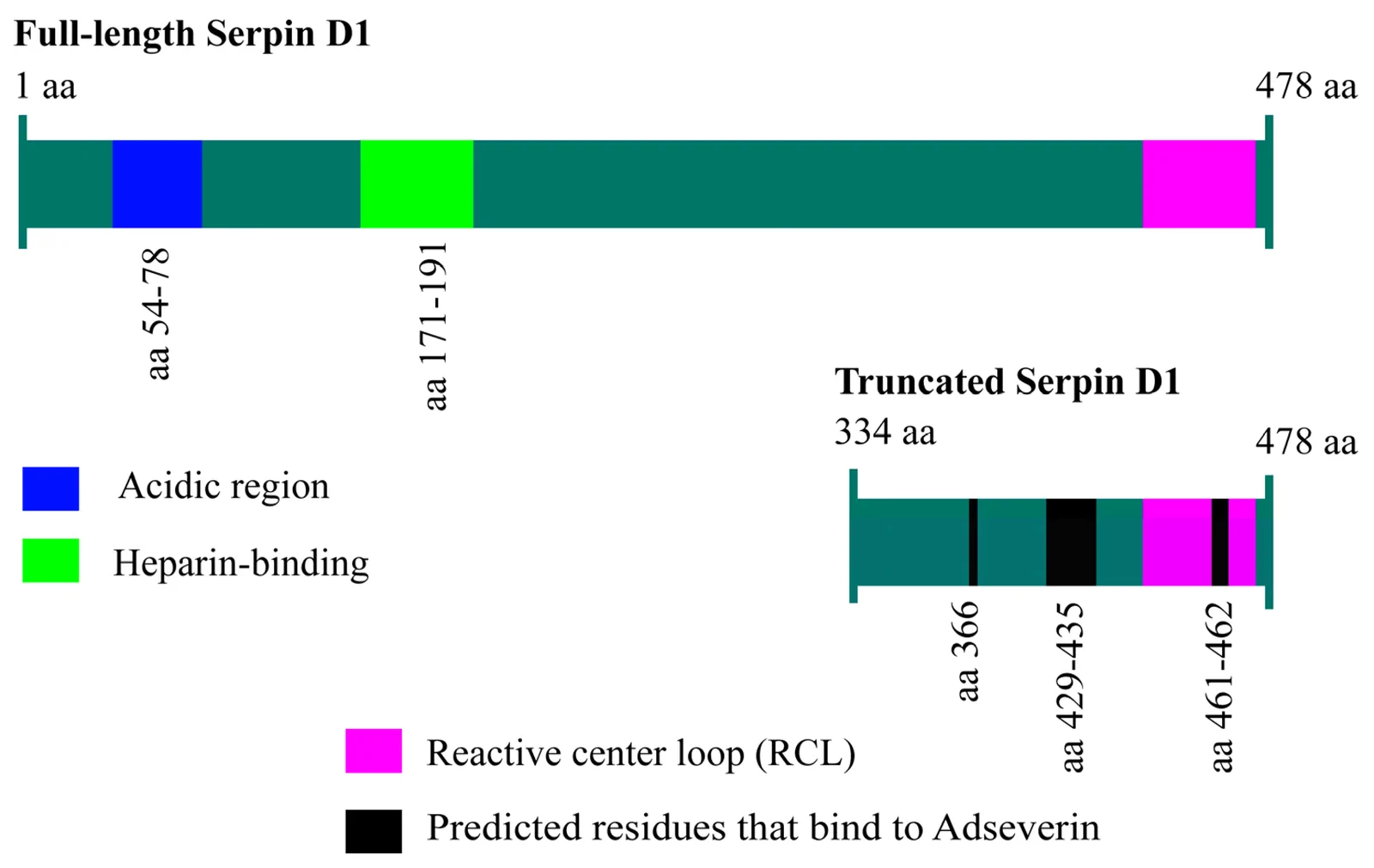
Schematic comparison of full-length and truncated Serpin D1 constructs. The truncated Serpin D1 variant lacks the N-terminal region containing the heparin-binding domain but retains the C-terminal reactive center loop (RCL), which is critical for the inhibitory activity of Serpin D1.

We found by RT-PCR, immunoblotting and immunoprecipitation that the *Serpind1* expressed in mouse osteoclasts is alternatively transcribed and does not contain exons 1 and 2, as shown earlier [25]. Serpin D1 detected in Ads immunoprecipitates prepared from osteoclast lysates was ~30 kDa, which is expected based on the predicted size of the osteoclast-specific, alternatively transcribed *Serpind1* transcript. The predicted structure of the truncated Serpin D1 is shown in Figure 10.

We considered the possibility that CRISPR deletion in some of the experiments that we conducted may have affected regulatory elements that are independent of Serpin D1 expression and that may have affected experimental outcomes in which CRISPR was used. While CRISPR/Cas9-mediated gene editing can potentially affect local regulatory elements in addition to disrupting the target gene, several lines of evidence support a Serpin D1-dependent mechanism in the current manuscript. Targeting exon 2, which is not present in the osteoclast-expressed *Serpind1* transcript, did not affect the phenotype in three independent clones, whereas deletion of exon 3 produced a consistent phenotype in two independent clones. In addition, re-expression of Serpin D1 partially rescued the phenotype. Collectively these findings suggest that the observed effects are primarily attributable to loss of Serpin D1 function, although contributions from local genomic alterations cannot be completely excluded.

Arising from our data showing an important role for a truncated Serpin D1 variant in osteoclast formation, we examined the roles of exon 2 or exon 3 of *Serpind1* in osteoclastogenesis. Compared with WT osteoclasts, the expression levels of the osteoclast-specific marker genes *Acp5* (TRAcP), *Ctsk* and *Atp6v0d2*, and of *Nfatc1* (a transcription factor functionally linked to the control of osteoclast-specific genes), were markedly reduced in osteoclasts in which the *Serpind1* exon 3 was deleted. But using CRISPR/Cas9 to delete exon 2 of *Serpind1* in RAW264.7 cells, the removal of exon 2 did not alter the expression of the alternatively transcribed *Serpind1* at mRNA or protein levels. These data support our contention that full-length *Serpind1* is not expressed in mouse osteoclasts. Further, in osteoclasts derived from the exon 3-deleted cells, we did not detect *Serpind1* mRNA in RANKL-treated cells. Taken together these data indicate that the alternatively transcribed *Serpind1* may play a role in osteoclast differentiation at inflamed sites (e.g., periodontitis, rheumatoid synovium). This notion is supported by our PLA data showing colocalization of Serpin D1 and Ads in the subcortical regions of RANKL-induced pre-fusion osteoclasts and multinuclear osteoclasts, and by our observations that the alternatively transcribed Serpin D1 enhances the severing activity of Ads in vitro. Future research might consider the nature of the inflammatory context in which the truncated Serpin D1 enhances osteoclast formation, and the increase of the concentrations of Serpin D1 needed to mediate these processes.

Computational modeling revealed that the majority of contact points in Serpin D1 are located towards the C-terminus, predominantly within exons 3 to 5, which harbor the highest density of potentially interacting residues with Ads. Serpin D1 is an extracellular, plasma serine protease that specifically functions as a thrombin and chymotrypsin inhibitor [46,47] and is one of the key regulators of the fibrinolysis and coagulation pathways [48]. Notably, serpin family members exhibit highly conserved secondary structures. A reactive center loop interacts with the protease active site to inhibit protease activity, which could also provide a docking site for Ads when it is activated by Ca^2+^. In this context, we used ratiometric imaging of fura-2 to estimate [Ca^2+^]_i_. The formation and disappearance of [Ca^2+^]_i_ loci or clusters after RANKL addition to WT cells indicated that cluster formation was a transient event but was not manifest in the *Serpind1* KO (E3) cells. Notably, during the initial stages of cell fusion in RANKL-induced osteoclasts, there is increased likelihood of the generation of Ca^2+^ transients [49], some of which may be restricted to cell adhesions such as podosomes [50] or other types of cell adhesions.

An interesting feature of the data set presented here is that Serpin D1 could influence Ca^2+^ signaling and thereby affect Ads-mediated actin severing. While the mechanistic relationship between these processes is currently undefined, Serpin D1 appears to function as a modulator of the Ca^2+^ sensitivity of Ads. In addition, Serpin D1 could play a role in the upstream regulation of Ca^2+^ signaling dynamics. While our data provides limited mechanistic insight on how Ads may regulate Ca^2+^ signaling, as Ads (and the structurally related actin severing protein gelsolin) exhibit Ca^2+^-dependent actin severing activity, we consider that Serpin D1 affects the Ca^2+^ sensitivity of Ads. The structural features that we describe of a specific interaction of Serpin D1 with Ads, which involve a possible docking site in the reactive center loop of Serpin D1, suggest that Ads-Serpin D1 binding is activated by Ca^2+^. This process also promotes actin severing. Thus, in early processes of cell fusion required for osteoclast formation, increased [Ca^2+^]_i_ in WT cells may contribute to the binding of Serpin D1 to Ads, further enhancing actin severing and cell fusion.

**Figure 11:**
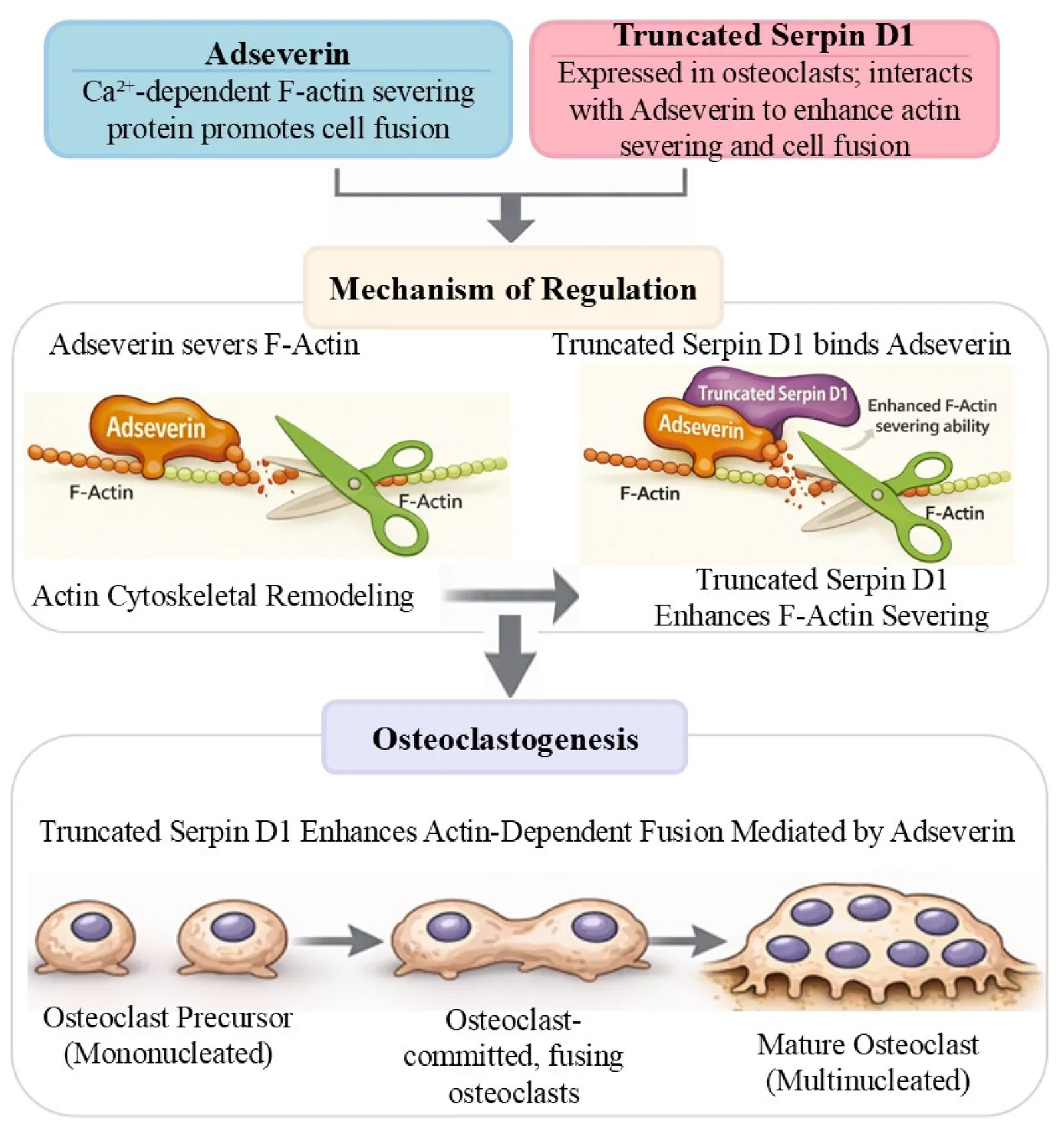
Proposed model illustrating the interaction between Serpin D1 and Ads during osteoclastogenesis. Ads severs actin filaments (F-actin) in the cell cortex of monocyte precursor cells. Binding of truncated Serpin D1 enhances Ads-mediated F-actin severing, a process that promotes actin-dependent fusion of mononuclear osteoclast precursors.

Our finding of an interaction between Serpin D1 and Ads requiring increased Ca^2+^ fits with the ability of Ads to undergo Ca^2 +^-dependent allosteric activation and interaction with actin filaments through domain 2 in its N-terminus [51]. Collectively, in view of the data presented here, we propose a model that focusses on the interaction between Serpin D1 and Ads during osteoclastogenesis. Ads severs actin filaments in the cell cortex, a process that is critical for fusion of monocytes to enable osteoclast formation. The binding of the osteoclast-associated, truncated Serpin D1 with Ads enhances actin filament severing, which promotes monocyte fusion and the generation of osteoclasts (Figure 11).

## 5. Conclusions

We suggest that an intracellular, truncated Serpin D1 variant plays a role in the activation of Ads and in the actin severing functions of this protein, which are important for osteoclastogenesis. Conceivably, the truncated Serpin D1 isoform, through its interactions with Ads to regulate actin severing, helps to mediate the formation of osteoclasts at inflamed sites.

## Figures and Tables

**Table 1:** Proteins immunoprecipitated with Ads antibody from RAW264.7 cells pre-treated with RANKL and TNF-α.

Proteins Identified by Mass Spectrometry		No. of Peptides Identified (*p* < 0.01)	Osteoclast-Associated Studies
Protein Name	Abbrev.		
Dedicator of cytokinesis protein 7	Dock7	31	[35,36]
Ads (I.P. targeted protein)	Scin	30	[17,18,19,29,37]
Actin, cytoplasmic 1	Actb	29	-
Isoform 2 of Gelsolin	Gsn	28	-
GTP-binding nuclear protein Ran	Ran	5	-
Actin-related protein 2	Actr2	4	[38,39]
Four and a half LIM domains protein 3	Fhl3	4	-
Cofilin-1	Cfl1	4	[40,41,42]
Heparin cofactor 2	Serpin D1	2	[25]

**Table 2:** Residue-level contacts between Serpin D1 (ligand) and Adseverin (receptor).

Receptor Secondary Structure *	Receptor Residue	Receptor Residue Number	Ligand Secondary Structure	Ligand Residue	Ligand Residue Number
H	ARG	187	S	ASN	366
S	VAL	210	S	ASN	366
S	GLU	212	S	ASN	366
S	GLN	207	-	THR	429
S	GLN	207	-	GLN	430
S	GLN	207	-	ALA	431
S	GLU	173	-	ALA	432
S	TYR	175	-	ALA	432
S	GLN	207	-	ALA	432
S	ILE	209	-	ALA	432
-	PRO	217	-	ALA	432
-	PRO	217	-	ALA	433
H	SER	218	-	ALA	433
H	GLU	219	-	ALA	433
-	SER	215	-	THR	435
-	GLU	216	-	THR	435
-	PRO	217	-	THR	435
H	SER	218	-	THR	435
H	ARG	198	-	HIS	461
H	ASN	200	-	ARG	462
H	GLY	35	-	ARG	462

* H—Alpha Helix, S—Beta Sheet.

## Data Availability

All materials that are of a non-commercial nature (i.e., cells) will be made available to interested scientists, along with any specific data sets that can be described in sufficient detail including the relevant information.

## Supplementary Materials

The following supporting information can be downloaded at https://www.mdpi.com/article/10.3390/cells15171607/s1. Figure S1: Western blot analysis of Serpin D1 in osteoclasts derived from specific cell samples indicated above the blot. Figure S2: BAPTA-AM treatment reduced osteoclast fusion; Figure S3: Full-length Serpin D1 binding with Ads; Figure S4: Physiological significance of actin severing enhancement by Serpin D1; Figure S5: Characterization of *Serpind1* exon 2 knockout mice; Figure S6: Attempted generation of homozygous *Serpind1* floxed mice; Table S1: PCR primers; Tables S2 and S3: Detailed amino acid residue and atomic-level contact data are provided; Table S4: Micro-CT analysis of *Serpind1* heterozygous KO mice; Videos S1 and S2: Four contact points located in exon 3 to exon 5 of *Serpind1* (amino acids 334-478) were identified. Videos S3–S8: Imaging by confocal microscopy and optical sectioning.

## Abbreviations

Ads	adseverin
CRISPR/Cas9	clustered regularly interspaced short palindromic repeats/CRISPR-associated protein 9
E2	exon 2-targeted
E3	exon 3-targeted
E3C2	exon 3 deletion, clone 2
E3C5	exon 3 deletion, clone 5
FL	full-length
GST	glutathione S-transferase
HCII	heparin cofactor II
KO	knockout
M-CSF	macrophage-colony stimulating factor
Nfatc1	nuclear factor of activated T cells 1
PMSF	phenylmethylsulfonyl fluoride
RANKL	receptor activator of nuclear factor kappa-B ligand
RIPA	radioimmunoassay precipitation assay
RT-qPCR	reverse transcriptase-(quantitative) polymerase chain reaction
TRAcP	tartrate-resistant acid phosphatase
TNF-α	tumour necrosis factor alpha
WT	wild-type
