# Supplementary material for: A Truncated Serpin D1 Variant Regulates Adseverin-Dependent Actin Severing and Is Required for Osteoclastogenesis
Source: Cells. 2026 Sep 3;15(17):1607. doi: 10.3390/cells15171607 (PMC13565667; doi:10.3390/cells15171607)

**Supplementary Figure S1.** Western blot analysis of Serpind1 in osteoclasts derived from specific cell samples indicated above the blot. Note the presence of full-length Serpind1 in all cell preparations and of the truncated Serpind1 in osteoclasts derived from wild type mice, from the homozygous KO mice and from the E3C2 Serpind1 clones.

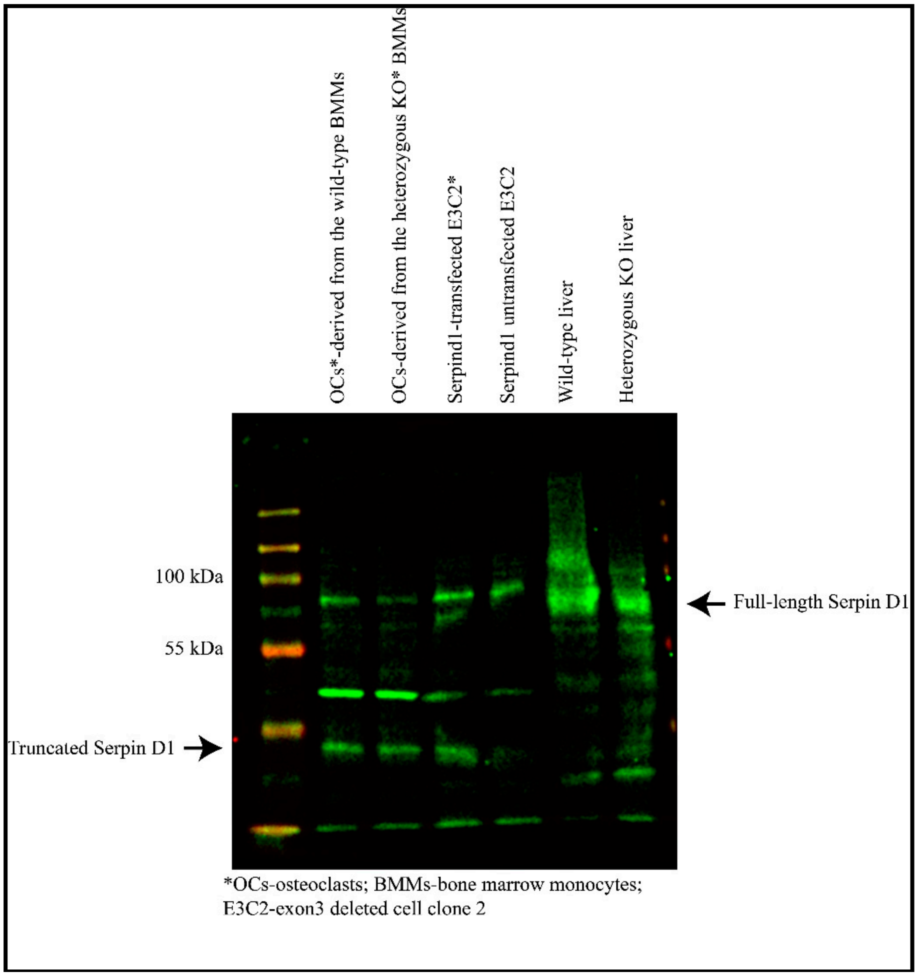

Supplement: Supplementary file 1 [file cells-15-01607-s001.zip › August 2026 Supplementary Figure S1 and Legend.pdf]
