# Supplementary material for: A Truncated Serpin D1 Variant Regulates Adseverin-Dependent Actin Severing and Is Required for Osteoclastogenesis
Source: Cells. 2026 Sep 3;15(17):1607. doi: 10.3390/cells15171607 (PMC13565667; doi:10.3390/cells15171607)
Supplement: Supplementary file 1 [file cells-15-01607-s001.zip › August 2026 Supplementary Figure S2 and Legend.pdf]

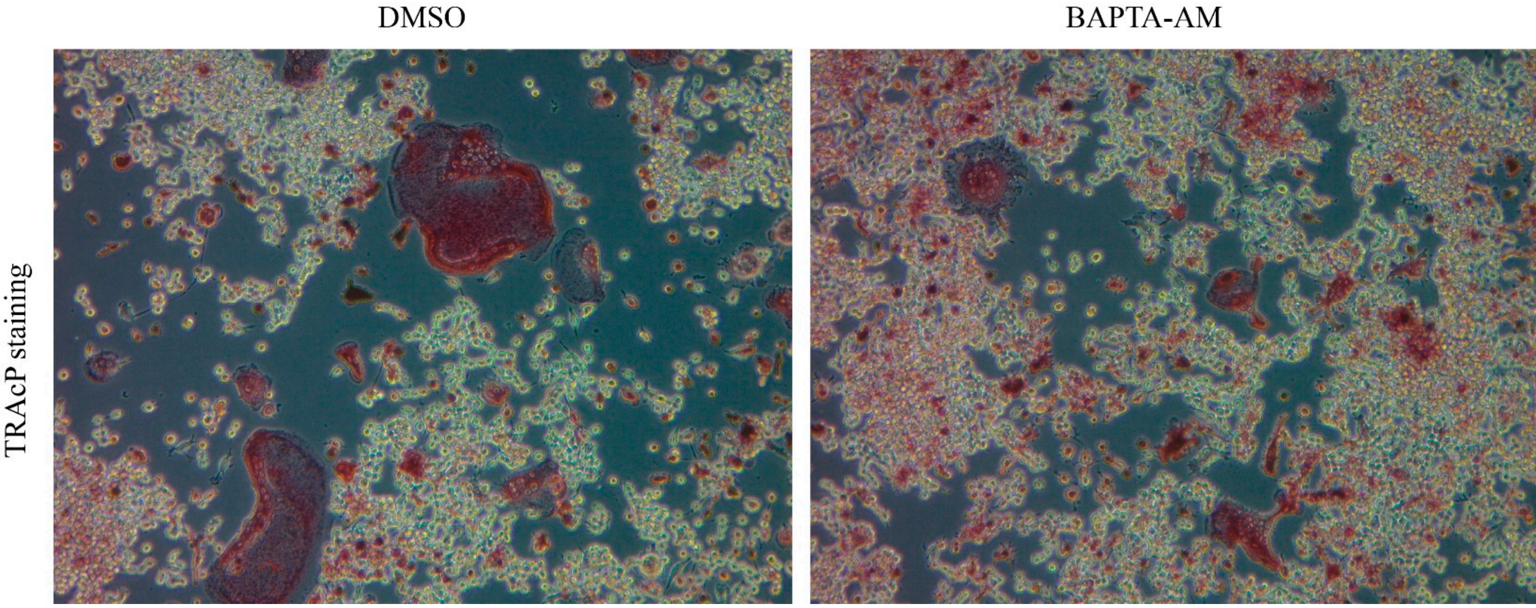

Supplementary Figure S2. BAPTA-AM treatment reduced osteoclast fusion. RAW264.7 cells were treated with RANKL for 3 days to induce pre-fusion osteoclasts. Cells were then incubated overnight with BAPTA-AM or DMSO, fixed with paraformaldehyde, and TRAcP-stained. Quantification of the proportion of multinucleated, TRAcP-positive cells from 3 different cultures revealed that BAPTA-AM reduced cell diameter (10-fold; $p<0.05$; Student's t-test), indicating that this treatment inhibited the formation of osteoclasts through cell fusion.
