# Supplementary material for: A Truncated Serpin D1 Variant Regulates Adseverin-Dependent Actin Severing and Is Required for Osteoclastogenesis
Source: Cells. 2026 Sep 3;15(17):1607. doi: 10.3390/cells15171607 (PMC13565667; doi:10.3390/cells15171607)
Supplement: Supplementary file 1 [file cells-15-01607-s001.zip › august 2026 Supplementary Figure S3 and legend.pdf]

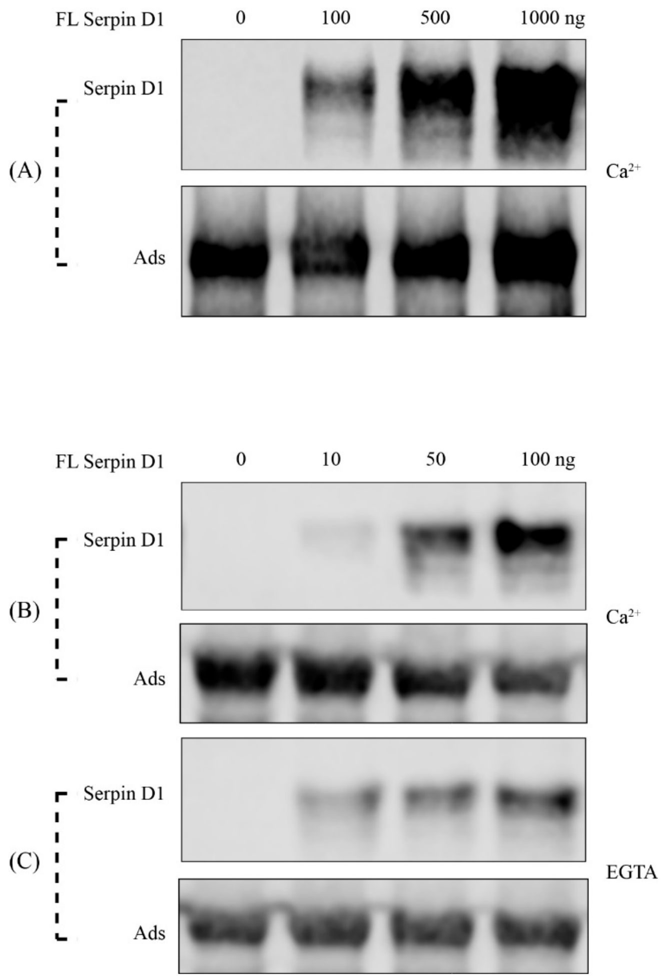

Supplementary Figure S3. Full-length Serpind1 binding with Ads. (A) GST-tagged recombinant Ads on glutathione Sepharose 4B beads was used to pull down full-length (FL) Serpind1 in a buffer containing 200 μM Ca^{2+}. The amount of pulled down Serpin D1 was influenced by the abundance of the initial Serpind1 (0-1000 ng) with which GST-Ads beads were incubated. The impact of Ca^{2+} on the binding of Serpind1 and Ads in vitro was assessed in buffers containing Ca^{2+} (B) or EGTA (C). These experiments indicated that the binding of Serpind1 to Ads is Ca^{2+}-dependent. Analysis of replicate samples (n=3) showed that compared with controls (buffer containing 200 μM Ca^{2+}), there was 55% (p<0.0.05) less Serpind1 binding to Ads in reactions conducted in a buffer containing 1 mM EGTA, a Ca^{2+} chelator (4B,C). All experiments were independently performed three times. Statistical analysis was performed using one-way ANOVA followed by Tukey's multiple comparisons test. The figures show representative set of results from single experiments. In the absence of Serpin D1 bound to beads (0 Serpin D1), there was negligible binding of Ads.
