# Supplementary material for: A Truncated Serpin D1 Variant Regulates Adseverin-Dependent Actin Severing and Is Required for Osteoclastogenesis
Source: Cells. 2026 Sep 3;15(17):1607. doi: 10.3390/cells15171607 (PMC13565667; doi:10.3390/cells15171607)

Supplemental Figure S4

Physiological significance of actin severing enhancement by Serpin D1

We conducted quantitative analysis of actin dynamics using pyrene actin fluorescence that was measured in time courses (see below). We examined whether there was enhanced actin severing activity of Ads over time that was associated with the truncated Serpind1. The full-length Serpin D1 was used as the positive control in this assessment. The panels in A (below) show individual experiments and the changes in pyrene actin fluorescence (means±standard deviations; n=3 replicates) are shown in panel B.

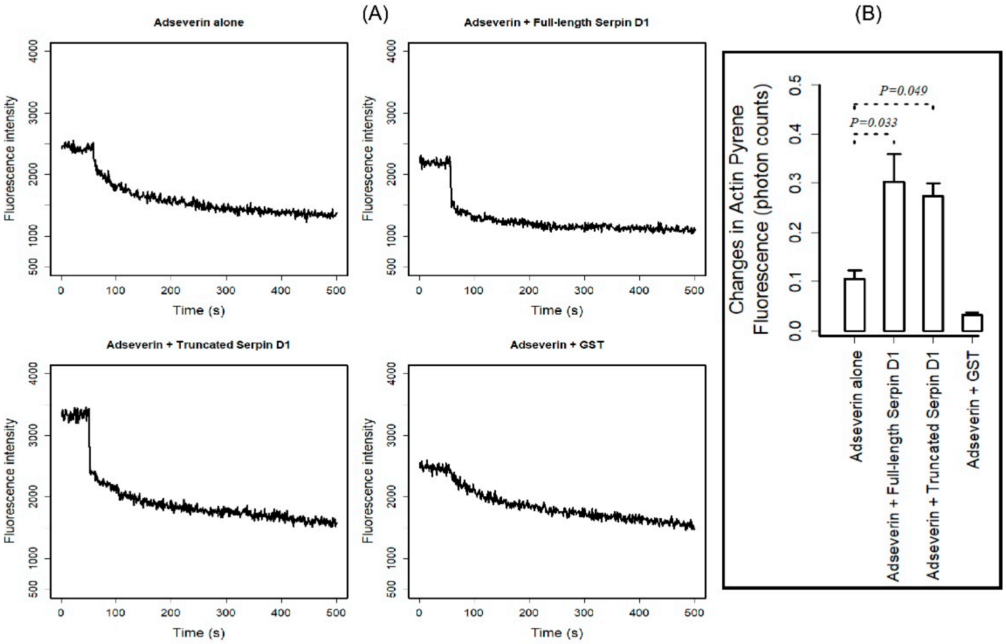

Supplement: Supplementary file 1 [file cells-15-01607-s001.zip › August 2026 Supplementary Figure S4.pdf]
