# Supplementary material for: A Truncated Serpin D1 Variant Regulates Adseverin-Dependent Actin Severing and Is Required for Osteoclastogenesis
Source: Cells. 2026 Sep 3;15(17):1607. doi: 10.3390/cells15171607 (PMC13565667; doi:10.3390/cells15171607)
Supplement: Supplementary file 1 [file cells-15-01607-s001.zip › August 2026 Supplementary Figure S5 and Legend.pdf]

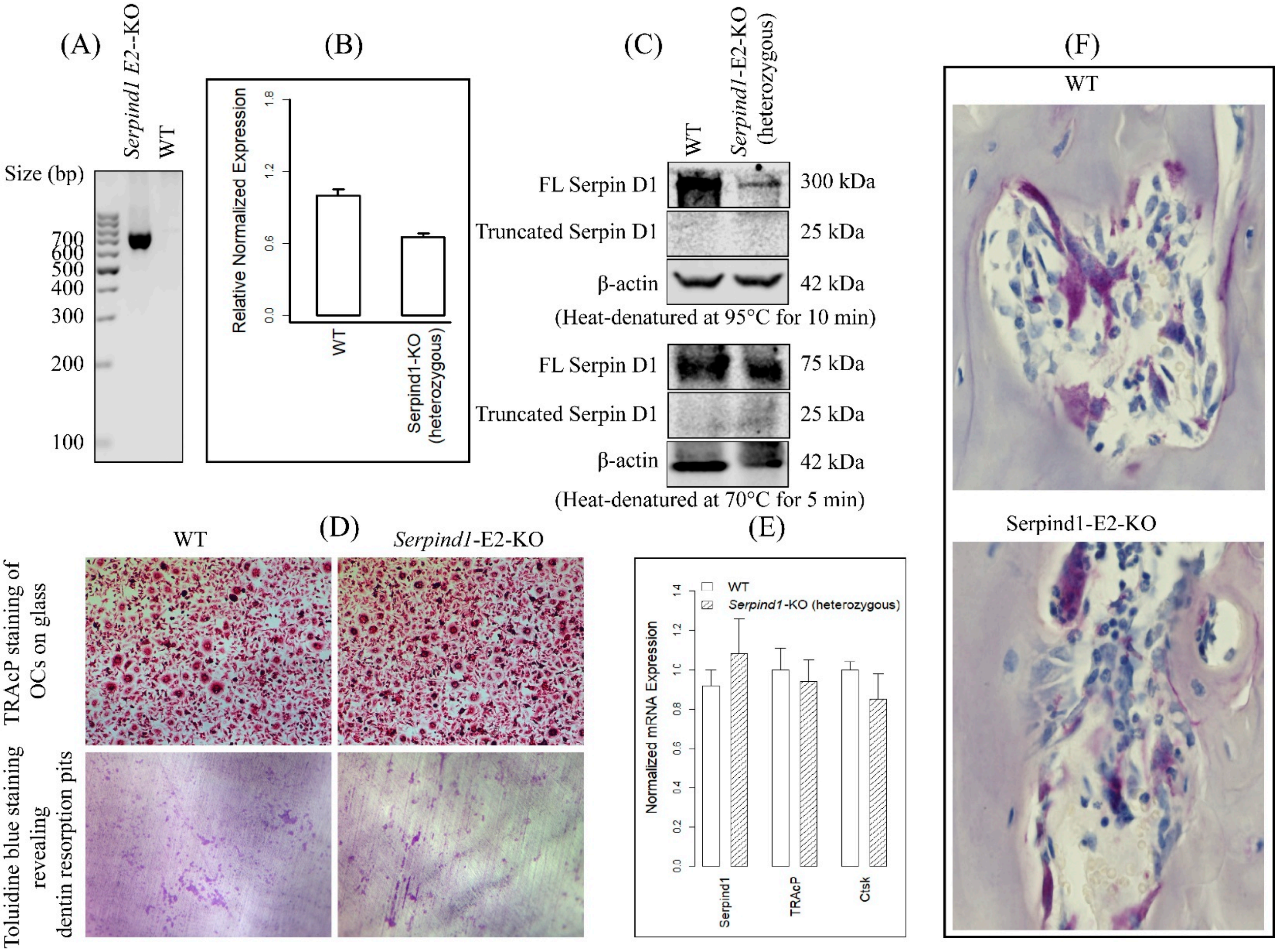

Supplementary Figure S5. Characterization of *Serpind1* exon 2 knockout mice. (A) Genotypic analysis of DNA extracted from ear notches identifying WT and *Serpind1* knockout mice. A 740-bp band amplified with a primer pair spanning the neomycin cassette and *Serpind1* identifies knockout mice, whereas no amplification indicates WT. (B) Liver tissues from two wild-type and two Serpind1 heterozygous mice were collected for RT-qPCR analysis to determine Serpind1 expression. RT-qPCR analysis of liver tissues showing reduced ($p<0.05$; Student's t-test) *Serpind1* expression in *Serpind1* exon 2 KO mice (heterozygous). Data are mean normalized expression levels± standard error of means from 3 replicates for each sample. (C) Western blot analysis confirming decreased Serpin D1 protein levels in liver tissues from *Serpind1* exon 2 KO mice (heterozygous). Liver lysates were heated either at 95°C for 10 min or at 70°C for 5 min prior to Western blot analysis. Full-length Serpin D1 migrated at ~300 kDa following heating at 95°C but migrated at ~75 kDa after heating at 70°C, suggesting there was heat-induced alteration of electrophoretic mobility under the more stringent denaturation conditions. Truncated Serpin D1 was not detected under either preparation method in liver tissues. (D) TRAcP staining of day-6 osteoclasts derived from WT and *Serpind1*-KO (heterozygous) mice cultured on glass or dentin slices (toluidine blue staining) demonstrating comparable RANKL-induced osteoclastogenesis and resorptive function. (E) RT-qPCR analysis showing similar expression levels of osteoclast-specific marker genes TRAcP and Ctsk, as well as Serpind1. Deletion of exon 2 did not alter Serpind1 expression in osteoclasts. Data are mean normalized mRNA expression levels from 3 replicates for each indicated sample. In vitro

osteoclastogenesis assays using primary bone marrow cells were independently performed four times. TRAcP staining and RT-qPCR analysis of osteoclast-specific marker gene expression showed a similar pattern, indicating no significant differences ($p>0.2$) in osteoclast formation or osteoclast-specific marker gene expression between cells derived from wild-type and Serpind1 heterozygous mice. Data show mean ± standard error of mean of 4 technically replicated measurements. (F) TRAcP and hematoxylin staining of distal tibial sections showing comparable numbers of TRAcP-positive multinucleated osteoclasts on trabecular bone surfaces in WT and *Serpind1*-KO (heterozygous) mice.
