# Supplementary material for: A Truncated Serpin D1 Variant Regulates Adseverin-Dependent Actin Severing and Is Required for Osteoclastogenesis
Source: Cells. 2026 Sep 3;15(17):1607. doi: 10.3390/cells15171607 (PMC13565667; doi:10.3390/cells15171607)
Supplement: Supplementary file 1 [file cells-15-01607-s001.zip › August 2026 Supplementary Figure S6 and legend.pdf]

Supplementary Figure S6. Attempted generation of homozygous Serpind1 floxed mice

To study the role of the truncated Serpind1 isoform in osteoclasts and the bone phenotypes associated with Serpind1-null osteoclasts, we generated LoxP-inserted mice in which exon 3 of Serpind1 was floxed. Exon 3 corresponds to the transcriptional start site of the truncated Serpind1 isoform that is specifically expressed in osteoclasts. These mice were intended to be crossed with a Cre recombinase line driven by the cathepsin K promoter to achieve conditional knockout of Serpind1 in osteoclasts.

Four pairs of heterozygous LoxP-floxed mice were used as breeders and produced a total of 76 pups. Ear notch samples were collected for genotyping using two pairs of primers designed to detect LoxP insertion at both the distal and proximal sites flanking exon 3. (PCR primers: Genotyping premiers. F3: 5'-ATGAGATGATACAAGAAAGGTGCC-3'paired with R5: 5'-GTGATTCTCTAGCATTTGGCAGGA-3'; F4:5'-TAAGCTATGCCTGTTTATTTCAGCC-3'paired with R6: 5'-GCAGACTGAGGCTATTCCTAAGAT-3'). Among the 76 pups, 27 were identified as wild type and 49 as heterozygous, while no homozygous LoxP mice were obtained. Sequencing confirmed that the lower PCR band observed in heterozygous mice corresponded to the wild-type Serpind1 allele. These results suggest that homozygous LoxP insertion is embryonic lethal, preventing the generation of the desired conditional knockout model.

Several enhancers and transcription factor binding sites are annotated within the intronic regions of Serpind1 and may have been disrupted by the LoxP insertion. In addition, the Pi4ka gene is in the same genomic region as Serpind1 but is transcribed in the opposite direction. Because null alleles of Pi4ka are reported to be embryonic lethal, disruption of Pi4ka expression by the LoxP insertion could contribute to the observed lethality. Alternatively, disruption of regulatory elements in this region could affect the expression of neighboring genes.

To investigate this possibility, we examined the expression levels of Serpind1 and Pi4ka. Serpind1 expression was assessed in liver tissue, and Pi4ka expression was analyzed in liver and kidney tissues. Using RT-qPCR, we compared Pi4ka expression between heterozygous and wild-type mice (n = 2 per genotype). The results showed that Serpind1 expression levels were similar between genotypes, whereas Pi4ka expression in heterozygous mice was approximately half of that observed in wild-type mice.

Representative genotyping results for 32 pups from heterozygous breeding using the F4/R6 primer pair (amplicon size mutant 202 bp, wild-type 134 bp) are shown below.

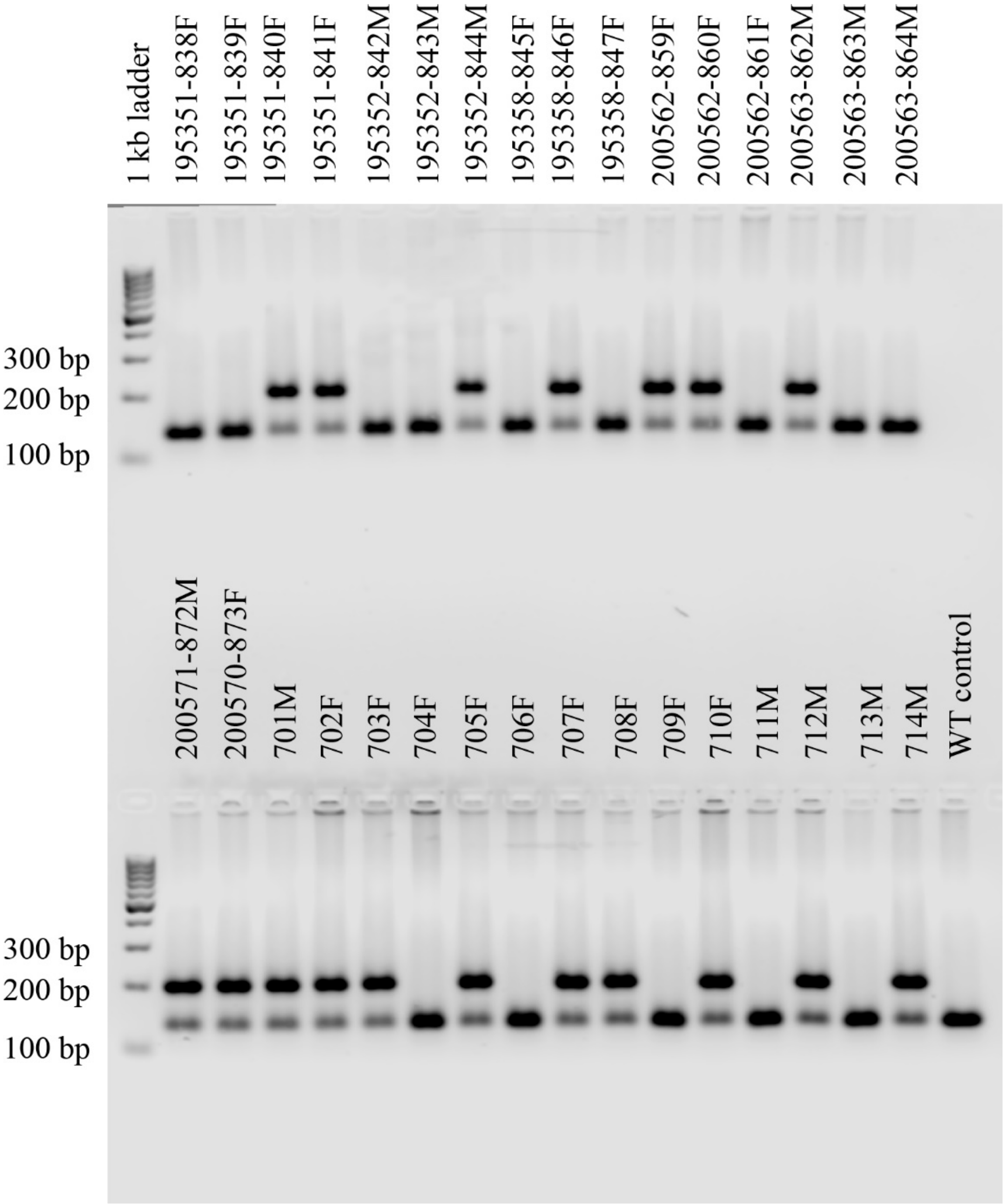
1 kb ladder
195351-838F
195351-839F
195351-840F
195351-841F
195352-842M
195352-843M
195352-844M
195358-845F
195358-846F
195358-847F
200562-859F
200562-860F
200562-861F
200563-862M
200563-863M
200563-864M
300 bp
200 bp
100 bp
200571-872M
200570-873F
701M
702F
703F
704F
705F
706F
707F
708F
709F
710F
711M
712M
713M
714M
WT control
300 bp
200 bp
100 bp
