# Supplementary material for: A Truncated Serpin D1 Variant Regulates Adseverin-Dependent Actin Severing and Is Required for Osteoclastogenesis
Source: Cells. 2026 Sep 3;15(17):1607. doi: 10.3390/cells15171607 (PMC13565667; doi:10.3390/cells15171607)
Supplement: Supplementary file 1 [file cells-15-01607-s001.zip › Supplementary Table S1 January 10 2026.pdf]

Supplementary Table S1. PCR primers

Gene	GenBank accession no.	Forward (F) and Reverse (R) primers (5'-3')		Primer location (exon)	Amplicon size (bp)	Reference
SERPIND1	NM_008223.4	F	ATCTCTGCCACACCACTGCT	1	532	
		R	CGGTAAAGGTTGAAGGCAAA	2		
		F	ATCTCTGCCACACCACTGCT	1	1,341	
		R	GGACCTCCACCAGGTTGTAA	4		
		F	GGCAAACAACCACATTCTG	2	443	
		R	GGACCTCCACCAGGTTGTAA	4		
		F	GAACTTGGGTGAATAAATTCCCAG	3 (nt1*)	336	
		R	GGACCTCCACCAGGTTGTAA	4		
		F	ATGACGCACAACCACAACTTCC	3 (nt30*)	307	
		R	GGACCTCCACCAGGTTGTAA	4		
		F	GAGTACGTAGGGGGCATCAG	3 (nt153*)	184	(Charles et al., 2012)
		R	GGACCTCCACCAGGTTGTAA	4		
Scinderin (SCIN)	NM_001146196.1	F	CCTATGGTGACTTTTACGTCGG	1	116	(Hassanpour et al., 2014)
		R	CTCATCCTGGGAACACTCCTT	2		
TRAcP (ACP5)	NM_001102405	F	ACGGCTACTTGCGGTTTCA	5	138	
		R	TCCTTGGGAGGCTGGTCTT	5		
Cathepsin K (CTSK)	NM_007802	F	GAAGAAGACTCACCAGAAGCAG	2	102	(Wang et al., 2008)
		R	TCCAGGTTATGGGCAGAGATT	3		
NFATC1	NM_198429	F	TGAGGCTGGTCTTCCGAGTT	5	91	(Verron et al., 2010)
		R	CGCTGGGAACACTCGATAGG	5-6		
ATP6V0D2	NM_175406	F	CATCTCCAGACCACGGACTAT	2	154	
		R	AGGTGAGAAATGTGCTCAGGG	2		
GAPDH	NM_001289726.1	F	CCTTCCGTGTTCCTACCCC	5	131	(Wang et al., 2018)
		R	GCCCAAGATGCCCTTCAGT	6		
		F	CGACTTCAACAGCAACTCCCACTCTT	6	335	
		R	GGGTGCAGCGAACTTTATTGATGGTA	7		

*nt1, nt30, and nt153: forward primers initiating at the indicated nucleotide positions within Serpind1 exon 3.
