# Supplementary material for: A Truncated Serpin D1 Variant Regulates Adseverin-Dependent Actin Severing and Is Required for Osteoclastogenesis
Source: Cells. 2026 Sep 3;15(17):1607. doi: 10.3390/cells15171607 (PMC13565667; doi:10.3390/cells15171607)
Supplement: Supplementary file 1 [file cells-15-01607-s001.zip › Supplementary Table S4.pdf]

Table S4. Micro-CT analysis of *Serpind1* heterozygous KO mice

		WT		KO		*P*-value
Trabecular		Mean	S.D.	Mean	S.D.	
Tissue volume	TV (mm^3)	1.657	0.146	1.626	0.174	0.799
Bone volume	BV (mm^3)	0.219	0.095	0.213	0.095	0.926
Percent bone volume	BV/TV (%)	12.954	4.921	12.823	4.804	0.971
Tissue surface	TS (mm^2)	9.986	0.572	9.927	0.91	0.917
Bone surface	BS (mm^2)	17.771	5.967	17.153	5.445	0.887
Bone surface / volume ratio	BS/BV (1/mm)	85.202	13.015	85.058	12.356	0.988
Trabecular pattern factor	Tb.Pf (1/mm)	28.271	9.653	28.023	10.158	0.973
Trabecular thickness	Tb.Th (mm)	0.482	0.046	0.496	0.005	0.703
Trabecular number	Tb.N (1/mm)	2.631	0.836	2.531	0.783	0.868
Trabecular separation	Tb.Sp (mm)	0.219	0.025	0.229	0.219	0.562
Bone mineral density	BMD (g/cm^3)	0.223	0.045	0.223	0.041	0.999
Cortical bone						
Bone volume (shrink)	BV (mm^3)	1.056	0.107	1.032	0.107	0.761
Bone volume (donut)	BV (mm^3)	1.055	0.106	1.029	0.104	0.733
Percent bone volume (shrink)	Percent bone volume	40.95	0.737	41.244	0.936	0.639
Percent bone volume (donut)	Percent bone volume	65.727	2.162	65.606	1.982	0.937
Cross-sectional thickness (shrink)	Cs.Th (mm)	0.163	0.015	0.167	0.008	0.626
Cross-sectional thickness (donut)	Cs.Th (mm)	0.163	0.015	0.165	0.007	0.787
Bone mineral density	BMD(g/cm^3)	1.242	0.026	1.204	0.02	0.058
2D slices						
Mean thickness-top	Th (mm)	0.182	0.012	0.183	0.007	0.889
Mean thickness-middle	Th (mm)	0.194	0.015	0.192	0.012	0.806
Mean thickness-bottom	Th (mm)	0.209	0.017	0.206	0.007	0.755
